# Characterizing the *Escherichia coli* O157:H7 Proteome Including Protein Associations with Higher Order Assemblies

**DOI:** 10.1371/journal.pone.0026554

**Published:** 2011-11-07

**Authors:** Rembert Pieper, Quanshun Zhang, David J. Clark, Shih-Ting Huang, Moo-Jin Suh, John C. Braisted, Samuel H. Payne, Robert D. Fleischmann, Scott N. Peterson, Saul Tzipori

**Affiliations:** 1 J. Craig Venter Institute, Rockville, Maryland, United States of America; 2 Division of Infectious Diseases, Cummings School of Veterinary Medicine, Tufts University, North Grafton, Massachusetts, United States of America; The Scripps Research Institute, United States of America

## Abstract

**Background:**

The recent outbreak of severe infections with Shiga toxin (Stx) producing *Escherichia coli* (STEC) serotype O104:H4 highlights the need to understand horizontal gene transfer among *E. coli* strains, identify novel virulence factors and elucidate their pathogenesis. Quantitative shotgun proteomics can contribute to such objectives, allowing insights into the part of the genome translated into proteins and the connectivity of biochemical pathways and higher order assemblies of proteins at the subcellular level.

**Methodology/Principal Findings:**

We examined protein profiles in cell lysate fractions of STEC strain 86-24 (serotype O157:H7), following growth in cell culture or bacterial isolation from intestines of infected piglets, in the context of functionally and structurally characterized biochemical pathways of *E. coli*. Protein solubilization in the presence of Triton X-100, EDTA and high salt was followed by size exclusion chromatography into the approximate M_r_ ranges greater than 280 kDa, 280-80 kDa and 80-10 kDa. Peptide mixtures resulting from these and the insoluble fraction were analyzed by quantitative 2D-LC-nESI-MS/MS. Of the 2521 proteins identified at a 1% false discovery rate, representing 47% of all predicted *E. coli* O157:H7 gene products, the majority of integral membrane proteins were enriched in the high M_r_ fraction. Hundreds of proteins were enriched in a M_r_ range higher than that predicted for a monomer supporting their participation in protein complexes. The insoluble STEC fraction revealed enrichment of aggregation-prone proteins, including many that are part of large structure/function entities such as the ribosome, cytoskeleton and O-antigen biosynthesis cluster.

**Significance:**

Nearly all *E. coli* O157:H7 proteins encoded by prophage regions were expressed at low abundance levels or not detected. Comparative quantitative analyses of proteins from distinct cell lysate fractions allowed us to associate uncharacterized proteins with membrane attachment, potential participation in stable protein complexes, and susceptibility to aggregation as part of larger structural assemblies.

## Introduction

Enterohemorrhagic *E. coli* (EHEC) are a group of bacteria containing many serotypes that are responsible for outbreaks of bloody diarrhea often leading to serious systemic complications such as the hemolytic uremic syndrome (HUS) and neurological abnormalities which in severe cases can be fatal [1]. These complications are attributed to Shiga toxins (Stx), shared with *Shigella dysenteriae* and acquired via horizontal gene transfer through phages [2]. The most common cause of outbreaks and sporadic cases of bloody diarrhea and HUS in the US are strains of the serotype O157:H7. The recent outbreak in Europe highlights the fact that Stx producing *E. coli* (STEC) serogroups, other than *E. coli* O157:H7, unable to form attachment and effacement lesions in the colon which are characteristic for EHEC [3], are also capable of causing severe systemic illness in humans [4]. STEC is defined by the CDC as a pathogenic *E. coli* isolate/strain expressing an active Shiga toxin and causing diarrhea or more severe disease symptoms. STEC strain 86-24 caused an outbreak of hemorrhagic colitis with a high mortality rate in 1986 [5] and was shown to produce only Shiga toxin 2 (Stx2) [6]. While epidemiological data suggest that Stx2 producers are more frequently associated with HUS than Stx1 producers, it is less clear whether Stx2 or Stx1 and Stx2 producers are more prevalent among strains causing severe disease complications [7], [8]. Pig is the only species besides human naturally susceptible to the systemic effects of Stx, and Stx2 producers in particular have caused severe neurologic symptoms, beyond bloody diarrhea, in the piglet model [9].

Shotgun proteomics is a term used for experiments in which complex protein samples are digested into peptide fragments followed by one or several dimensions of liquid chromatography (LC) to separate the peptides and tandem mass spectrometry (MS) to identify these peptides via mapping of peptide-spectral matches (PSMs) back to the proteins of origin [10], [11]. Mapping the PSMs is facilitated by powerful computational algorithms such as Mascot [12], Protein/Peptide Prophet [13] and InsPecT [14] and availability of accurate protein sequence databases derived from annotated genomes. 2D-LC-MS/MS refers to two orthogonal dimensions of peptide separations prior to tandem MS (also termed MS^2^), the latter one typically performed with a nano-flow C_18_-reversed phase capillary column. Peptide separation methods continue to evolve and include specialized applications using affinity and size exclusion chromatography (SEC) and biphasic columns [15]. The diversity of methods developed for protein quantification from shotgun proteomics data is considerable, as recently reviewed [16]. Among label-free quantification techniques, the major ones are based on MS^1^ normalized peak intensity measurements, such as the accurate mass and time tag approach [17], and MS^2^ based spectral counting techniques, such as emPAI [18] and APEX [19]. Due to experimental variability, e.g. in the peptide LC elution times in highly complex mixtures and because of stochastic sampling and ion suppression effects during MS^1^ and MS^2^ scans, quality control metrics are important to maintain reproducible conditions across all analyses compared in a given experiment [20], [21]. To compute the probability of detection for each peptide theoretically present in a shotgun proteomics dataset, the APEX method employs machine learning techniques [19]. The computations result in peptide/protein O_i_ values (correction factors that define detection probabilities) that are integrated into an equation to normalize spectral count values. This method provides added flexibility in that it allows quantitative comparisons of the same protein across datasets (samples) and of different proteins in the same dataset. To validate the latter analysis feature, we recently assessed the stoichiometry of several *Shigella dysenteriae* protein complexes with known subunit ratios experimentally and obtained CVs of less than 20% for several soluble intracellular complexes [22].

Shotgun proteomics experiments without fractionation on the subcellular or protein level do inherently not produce information on protein assembly in larger entities (such as complexes and organelles), co-fractionation, solubility, membrane attachment and subcellular localization. Non-denaturing protein fractionation performed prior to 2D-LC-MS/MS affords the opportunity to evaluate patterns of protein distributions in a distinct fraction and among several fractions. To our knowledge, only two publications have described the use of SEC to separate proteins via M_r_ prior to shotgun 2D-LC-MS/MS analysis. The human mammary epithelial cell proteome [23] and the human liver tissue [24] were profiled, in each case resulting in expanded proteome coverage with ∼1,600 protein identifications. These data were not examined with the purpose to establish patterns of protein fractionation. Although SEC needs to be used in combination with light scattering and refractive index measurements to determine reliable M_r_ values for protein complexes [25], the technique is quite useful to estimate whether the native M_r_ of a protein corresponds to a monomer, a homo-oligomer or a larger structural entity involving multiple proteins, phospholipids, glycans or ribonucleoproteins. Proteomic profiling of soluble *versus* insoluble cell lysate fractions was applied to *Escherichia coli* strain MG1655, resulting in 1,147 protein identifications with enrichment of some in the insoluble fraction [26]. Insoluble *E. coli* lysate fractions have also been studied to assess the propensity of proteins to form aggregates [27], [28]. The aforementioned studies were elegant but did not focus on robust global quantification of proteins comparing lysate fractions or correlation of data with protein participation in higher order complexes.

The proteomics literature on *E. coli* is extensive [29]. In addition to protein profiling by two-dimensional gel electrophoresis (2D-GE) and LC-MS/MS, studies employing large-scale affinity purification followed by mass spectrometry (AP-MS) have contributed to determine novel functional roles, interactions and networks of proteins [30], [31], [32]. The combined number of physical *E. coli* protein-protein interactions from these studies was ∼17,500. Single-cell *E. coli* proteomic and transcriptomic data revealed surprisingly low correlations of protein and mRNA copy numbers [33]. At least a third of the *in silico* predicted *E. coli* proteome remains to be functionally characterized. Even for proteins attributed to have a specific function, novel functions continue to emerge, e.g. the roles of elongation factor Tu in cell shape maintenance [34] and enolase as a component of the mRNA degradosome [35]. The first genome of an *E. coli* serotype O157:H7 strain was published by Perna *et al.* [2]. Remarkably, 1,387 new genes encoded in strain-specific clusters were discovered in *E. coli* O157:H7 compared to strain MG1655. Most of the lateral gene transfer appeared to have occurred by phage integration. A key pathogenicity island in EHEC is the locus of enterocyte effacement (LEE). Some LEE-expressed proteins contribute to intimate bacterial adherence to intestinal epithelial cells, and the resident type 3 secretion system (T3SS) injects effectors into the host cell cytoplasm [36]. *E. coli* O157:H7 strains also feature a repertoire of effectors whose genes are spread across the genome [37]. The literature pertaining to STEC and EHEC proteomes and their adaptations to the host environment is sparse. In addition to a study discovering novel T3SS effectors [37], protein-based responses to oxidative stress in a stress-sensitive *E. coli* O157:H7 strain were examined [38].

Two major objectives were pursued in the research described here. First, using fractionation on the protein level and shotgun proteomics, the intention was to profile the *E. coli* serotype O157:H7 (strain 86-24) proteome. Secondly, we systematically examined whether biochemically relevant information can be derived from cell lysate fractionations, taking advantage of existing information in databases for *E. coli* protein functions, localizations, large subcellular assemblies and biosynthetic and metabolic pathways. Instead of using only cultured bacteria to accomplish these objectives, we also processed bacteria directly from the large bowel of gnotobiotic piglets infected with *E. coli* O157:H7.

## Methods

### Ethics Statement

Experiments in piglets were performed to allow analysis of the *E. coli* O157:H7 proteome from an *in vivo* environment. Piglets were delivered by cesarian section and housed at the Division of Infectious Disease of Tufts University School of Veterinary Medicine in accordance with approved procedures of the Institutional Animal Care and Use Committee at Tufts University. The animal studies were specifically approved by this committee. The respective protocols were No. G770-6 (titled gnotobiotic piglet model of *E. coli* infection, date 4-24-2006) and No. G2009-52 (titled gnotobiotic piglet model of *E. coli* infection, date 5-4-2009).

### Bacterial cell growth and recovery from suspension culture and animal experiments

Cells of the *E. coli* O157:H7 strain 86-24 were isolated from *in vitro* cell cultures and *in vivo* animal experiments. Cells were grown *in vitro* in Luria-Bertani (LB) broth to stationary phase (OD_600_∼2.0) in a shaking incubator at 37°C, pelleted by centrifugation at 7,000×*g* for 10 min at 4°C, washed with a 20-fold volume of PBS and re-concentrated at 7,000×*g* for 15 min at 4°C. As for the animal experiments, several piglets developed diarrhea 18–30 h post-inoculation and were euthanized 3 to 5 days later. Bacterial cells (between 1×10^9^ and 2×10^10^ cells) were recovered from the piglets' gut contents, repeatedly washed with PBS and purified via density gradient centrifugation with an isotonic 65% Percoll solution at 14,500×*g* for 30 min at 4°C, as previously described [39]. Up to 2×10^10^ bacterial cells were re-suspended in 1 ml of a hypotonic lysis buffer (25 mM Tris-OAc, pH 7.8, 0.05% Triton X-100, 5 mM Na-EDTA, and protease inhibitors benzamidine and AEBSF in 1 mM concentrations). Chicken lysozyme (150 µg/ml) was added to initiate cell lysis, agitating the suspension at 20°C for 1 h followed by quick-freezing and storage at −80°C until further processing. We did not observe any contamination by pig proteins in bacterial lysates derived from piglet intestines, judged by analysis of the 500 most dominant spots in 2D gels, all of which were identified as STEC proteins.

### Fractionation of cell lysates

To complete cell lysis, disintegrate post-lysis aggregates and degrade nucleic acids, each thawed suspension was sonicated (ten 30-sec on/60-sec off cycles at amplitude 5; Misonex 3000 sonicator) on ice. After addition of DNAse I and RNAse at 5 µg/ml and 15 mM MgCl_2_, the suspension was gently agitated for 1 h at 20°C and centrifuged at 16,100×*g* for 30 min at 4°C. Supernatant and insoluble pellet fractions were retained. The pellet was re-suspended in 300 µl of a high salt solution (50 mM Tris-OAc, pH 7.8, 2.5 M NaBr and 5 mM Na-EDTA), agitated for 1 h at 20°C, and spun again at 16,100×*g* for 30 min at 4°C. This supernatant was added to the 1^st^ lysate supernatant. The pellet was briefly washed with a 10-fold excess of PBS, and the wash solution was discarded. Small aliquots of the insoluble pellet and the lysate supernatant were analyzed in 4–12%T Coomassie Brilliant Blue G-250 (CBB)-stained SDS-PAGE gels to assess protein recovery. Following concentration to ca. 2 mg/ml protein, 250 µl aliquots of the supernatant were applied to the analytical SEC column G3000-SWXL (7.8 mm×30 cm, 5 µm particle size, TOSOH Bioscience, USA) and separated at a flow rate of 0.8 ml/min using PBS supplemented with 0.01% Triton X-100 at pH 7.5. Calibration runs on the SEC column were performed using the following protein standards: thyreoglobulin (670 kDa), bovine IgG (158 kDa), ovalbumin (45 kDa), myoglobin (18 kDa) and cobalamin (1.7 kDa). Elution peaks detected at A_280_ were plotted against native M_r_ values of proteins (log M_r_ *versus* elution volume V_E_), permitting a rough calculation of M_r_ ranges for the V_E_ segments derived from the SEC fractionation of samples. Fractions were collected in 2 min increments and analyzed in CBB-stained 4–12%T SDS-PAGE gels; those corresponding to 6.8–8.8 min, 8.8–10.8 min and 10.8–14.8 min elution times contained nearly all protein solubilized from lysates and were recovered for further analyses. The fractions termed F1-s_SEC_, F2-s_SEC_ and F3-s_SEC_, respectively, from here on were devoid of lysozyme, which eluted in V_E_ segments beyond the 15 min mark due to its low M_r_. The insoluble lysate pellet termed F4-p from here on was not subjected to SEC, but protein aliquots containing ca. 60–100 µg protein were also prepared for LC-MS/MS.

### Iodixanol density gradient centrifugation of insoluble *E. coli* fractions

Bacterial lysates derived from *ca.* 4×10^9^ cells (500 µl) grown *in vitro* to stationary phase were subjected to hydrostatic pressure cycling in the Barocycler NEP3229 (PBI Inc., South Easton, MA), using 15 cycles of exposure to 35,000 psi over 55 sec and 15 psi over 5 sec. This lysis method is gentler than sonication and minimizes membrane denaturation and aggregate formation. The lysis buffer and nucleic acid digestions were identical to conditions described above, except the reduction of post-lysis salt concentrations with only 150 mM NaCl. Iodixanol step gradient layers were prepared with an Optiprep™ stock solution (Axis Shield, Oslo, Norway) containing 50% iodixanol in a 50 mM HEPES buffer, pH 7.8, supplemented with 125 mM sucrose, 0.5 M NaOAc and 5 mM MgCl_2_. An unfractionated lysate in an aliquot of *ca.* 500 µl was mixed with 800 µl Optiprep™ stock solution (40% w/v iodixanol concentration) followed by overlays with 33.3%, 25%, 16.7% and 8.3% iodixanol (v/v), each in a 1 ml volume. The sample was spun in a SW60 rotor at 50,000 rpm (257,000×g) for 3 h at 18°C. Turbid bands of non-solubilized material detected in the gradients were carefully aspirated followed by 10-fold dilution with PBS to recover the solid pellet fractions by centrifugation at 16,000×g for 45 min.

### Digestion of protein fractions with trypsin

Soluble and insoluble protein fractions were digested using a method termed filter-aided sample preparation (FASP) [40]. Briefly, pellets were re-suspended in 200 µl of 25 mM Tris-OAc (pH 7.8), 0.05% Triton X-100 and 5 mM EDTA. Soluble SEC fractions were transferred stepwise to and concentrated in a Microcon YM-10 membrane filter unit (10 kDa M_r_ cut-off; Millipore, Billerica, MA) and re-equilibrated in 200 µl of 25 mM Tris-OAc (pH 7.8), 0.05% Triton X-100 and 5 mM EDTA. To each sample, 20 µl of a 1 M DTT stock solution and 12 µl of a 10% SDS stock solution were added, and proteins were denatured by incubation for 3 minutes at 95°C. Room temperature-adjusted samples were transferred back into Microcon YM-10 units and subjected to proteolytic digestion with trypsin (trypsin/bacterial protein ratio of 1∶30 to 1∶50) at 20°C overnight [40] following alkylation. Each filtrate contained a complex peptide mixture, collected by centrifugation at 14,000×*g* for 40 minutes in the flow-through. Each Microcon YM-10 filter unit was rinsed twice with 50 µl of 500 mM NH_4_HCOO and once with 100 µl of 50 mM NH_4_HCO_3_. The rinses were added back to the respective protein digestion filtrate, which was then reduced to dryness.

### Strong cation exchange chromatography (SCX)

The lyophilized fractions (F1-s_SEC_, F2-s_SEC_, F3-s_SEC_ and F4-p) were subjected to peptide separation via SCX on a Polysulfoethyl-Aspartamide column (4.6 mm×50 mm, 5 µm particle size; Nest Group, USA). Each protein digestion mix was reconstituted in SCX buffer A (5 mM KH_2_PO_4_, 25% CH_3_CN, pH 3.0). Linear gradients with SCX buffer B (5 mM KH_2_PO_4_, 0.7 M KCl, 25% CH_3_CN, pH 3.0) were run at a flow rate of 0.5 ml/min: 10 min at 0% buffer B; increase of buffer B concentration to 20% over 30 min, 50% over 20 min and 100% over 5 min; maintain flow at 100% B for 5 min. Fractions were collected in 3 min increments. Fractions 1+2, 3−5, 6+7, 8, 9, 10, 11, 12, 13, 14, 15, 16+17 and 18+19 were collected, individually or in pools as indicated, and lyophilized overnight. Following reconstitution in 75 µl of 1% formic acid in 2% CH_3_CN, fractions with a high salt content (15, 16+17 and 18+19) were desalted using ZipTip C_18_ cartridges using a vendor-provided protocol (Millipore, Billerica, MA).

### Reversed phase C_18_ LC-MS/MS and database searches

Peptide mixtures contained in the 52 SCX fractions (13 fractions for the samples F1-s_SEC_, F2-s_SEC_, F3-s_SEC_ and F4-p each) were subjected to 2^nd^ dimension separation on a BioBasic C_18_ column (75 µm×10 cm; New Objective, Woburn, MA). Protein digestions derived from iodixanol gradient insoluble fractions contained *ca.* 25–50 µg protein and were directly analyzed by C_18_ LC-MS/MS. The instrument set-up of the nano-ESI LTQ linear ion trap mass spectrometer (LTQ; Thermo, San Jose, CA) coupled to an upfront Agilent 1100 solvent delivery system was described previously [41]. It was calibrated prior to each LC-MS/MS run with 200 nmol human [Glu^1^]-fibrinopeptide B (M.W. 1570.57), verifying that elution times with a CH_3_CN gradient varied less than 10% and that peaks representing ion counts had widths at half-height of <0.25 min, signal/noise ratios >200 and heights >10^7^. Briefly, loading a 25 µl sample volume was followed by peptide binding and wash steps on a C_18_ trapping cartridge at a flow rate of 0.01 ml/min for 13 min. Peptides were eluted from the C_18_ cartridge and separated on the C_18_ column with 53 min binary gradient runs from 97% solvent A (0.1% formic acid) to 80% solvent B (0.1% formic acid, 90% AcCN) at a flow rate of 350 nl/min. Eluates entered the nano-ESI source, and mass spectra were acquired starting at 10% solvent B. Spectra were acquired in automated MS/MS mode, with the top five parent ions selected for fragmentation in scans of the m/z range 300–1,500 and with a dynamic exclusion setting of 90 sec, deselecting repeatedly observed ions for MS/MS. All peptide fractions from a given sample were run consecutively on the LC-MS/MS system. The LTQ search parameters (+1 to +3 ions) included mass error tolerances of ±1.4 Da for peptide precursor ions and ±0.5 Da for peptide fragment ions. The search engine used for peptide identifications was Mascot v.2.3 (Matrix Science). Search parameters allowed one missed tryptic cleavage, and were set for carbamidomethyl-modifications of cysteine and oxidation of methionine residues as fixed and variable modifications, respectively. The protein sequence database for the *enterohemorrhagic E. coli* O157:H7 strain EDL933 genome was downloaded from the RefSeq dataset in NCBI to enable Mascot searches, because the genome sequence of strain 86-24 has not been published.

### Quantitative protein analysis using spectral counting (APEX)

Protein identifications derived from Mascot v.2.3 searches required at least one unique peptide with an e-value <0.1. Mascot search peptide false discovery rates (FDRs) were determined searching an *in silico* randomized *E. coli* O157:H7 EDL933 protein sequence database. The average FDR over 19 2D-LC-MS/MS experiments was 1.3%. Following file conversion into the ‘mzXML’ format, MS data were re-scored using the algorithms PeptideProphet™ and ProteinProphet™ [42], [43]. Prot.xml files were analysed using an open-source software termed the APEX quantitative proteomics tool v1.1 that we developed in 2008 [44]. Using a pre-defined set of MS analysis parameters and 30 physicochemical properties provided by the APEX tool (default settings), ARFF files for *O_i_* computations were generated for a set of 100 highly abundant bacterial proteins with M_r_ values >30 kDa (according to 2D gel data) to obtain a good representation of tryptic peptides. Observation of tryptic peptides in the training dataset were correlated with the 30 physicochemical peptide properties, resulting in the computation of *O_i_* values (the expected number of unique proteotypic peptides for a given protein *i*) for all protein sequences, in this case 5397 annotated proteins in the *E. coli* O157:H7 EDL933 sequence database. The equation to calculate APEX_i_ values from 2D-LC-MS/MS data includes the O*_i_* correction factors, probability scores for protein identification (p_i_) and spectral counts (n_i_) as variables: 
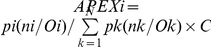
 Setting the protein FDR at 1%, only protein identified at a 99% confidence level were used for spectra counting. A factor of 2.5×10^6^, roughly the estimated number of protein molecules per *E. coli* cell, was used to normalize APEX scores as protein molecules per cell [45].

### Bioinformatics and statistics methods

Proteins were analyzed using various software tools to predict subcellular localizations, membrane attachment, molecular functions and pathway associations. Functions were derived from information in CMR (http://cmr.jcvi.org/), Swiss-Prot (www.uniprot.org/) and Ecocyc (http://ecocyc.org) databases, pathways examined in Ecocyc. For the *in silico* prediction of subcellular protein localizations, the software tools Cell PLoc (http://chou.med.harvard.edu/bioinf/Cell-PLoc) and PSORTb *v.*2.0 (www.psort.org/psortb/) were used. The algorithms of SignalP (export signal peptides), TMHMM (transmembrane domains, TMDs) and LipoP (lipid anchor motifs), all available at www.cbs.dtu.dk/, were used to detect motifs for export and membrane integration. BOMP was used for outer membrane (OM) β-barrel protein prediction (www.bioinfo.no/tools/bomp).

Data from eleven experiments pertaining to biological and technical replication with samples derived from cell growth *in vitro* were included in statistical analyses to assess quantitative patterns of protein fractionation. There were three biological cell culture replicates. Technical replication was performed prior to SEC and at the final step (C_18_ LC-MS/MS). Experimental reproducibility with respect to protein quantification was assessed calculating squared Pearson's Product Moment correlation coefficients (R^2^). Proteins not observed in both of the compared experimental pairs were not considered. Protein abundance data and associated annotations were imported into the MultiExperiment Viewer (MeV, http://www.tm4.org/mev/) [46] to perform statistical analysis and to cluster and visualize patterns of protein abundances across SEC fractions. Non-parametric statistical methods were applied to identify proteins that showed significant and consistent abundance changes between the SEC fractions under study. The Kruskal-Wallis test was performed to identify proteins with differential abundance among the three SEC fractions (F1-s versus F2-s versus F3-s). The two-sample Wilcoxon Rank Sum test was performed to find proteins differentially abundant comparing the collection of soluble protein fractions (F1-s+F2-s+F3-s) with the insoluble protein fraction (F4-p). Significant proteins from the Kruskal-Wallis test were re-imported into the MeV software to correlate the data with sequence-based, calculated M_r_s and M_r_s derived from protein complexes of a homo- or heteromultimeric nature that a given protein subunit is known to participate in in *E. coli*. Annotation data from UniProt and EcoCyc were considered, and the highest M_r_ value for a complex was chosen when variability in subunit composition was reported in the literature. Furthermore, hierarchical clustering (Euclidian distance and Pearson correlation) was performed using MeV to cluster ‘significant proteins’ based on similarity of their abundance patterns across all samples (n = 11) for each comparison. Clusters enriched in proteins that are part of multi-subunit complexes or larger subcellular assemblies were examined in depth.

## Results and Discussion

### Experimental approach

**Figure 1:**
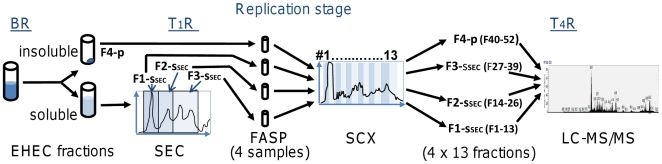
Experimental design combining two-dimensional LC-MS/MS with upfront fractionation of proteins derived from insoluble and soluble, SEC-separated cell lysate fractions. Abbreviations: EHEC, enterohemorrhagic *E. coli*; SEC, size exclusion chromatography; FASP, filter-aided sample preparation for digestion of complex protein mixtures; SCX, strong cation exchange chromatography; BR, biological replicate; T_1_R and T_4_R, technical replicate stages. The total number of SCX fractions subjected to LC-MS/MS sequentially was 52 in a given experiment.

Expanding a commonly used 2D-LC-MS/MS shotgun proteomics strategy for the analysis of bacterial lysates, we added a less frequently used soluble protein fractionation step, SEC (***Figure 1***). To establish a framework for comparisons of protein abundances, the computationally adjusted spectral counting method APEX that leverages quantitative comparisons of different proteins in a given dataset, in addition to relative comparisons of the same protein present in different fractions, was employed. Quantitative proteomic analysis was followed by data mining, including the correlation of enrichment of proteins in a distinct cell lysate fraction (soluble vs. insoluble) or a distinct SEC fraction (M_r_ ranges of >280 kDa vs. 280-80 kDa vs. 80-10 kDa) with its biochemical properties such as sequence-based and native M_r_ value, participation as a subunit in higher order protein assemblies, subcellular localization and attachment to or integration in phospholipid membranes. Since *E. coli* is one of the most extensively characterized prokaryotic organisms, plenty of information stored in various databases was available to assess the value of our approach and to build hypotheses. Based on the observed fractionation patterns of characterized *E. coli* proteins, we were able to infer similar properties for many uncharacterized *E. coli* O157:H7 proteins.

**Figure 2:**
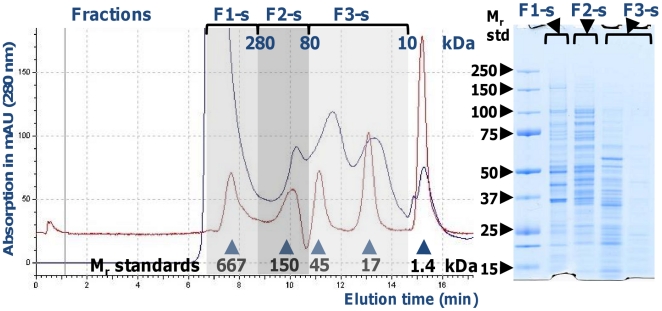
Size exclusion chromatography fractionating *E. coli* O157:H7 cell lysate proteins prior to 2D-LC-MS/MS. The fractions (F1-s, F2-s and F3-s) were eluted from the G3000-SWXL column in PBS and 0.01% Triton X-100 and pooled as shown in the chromatogram. The A_280_ profiles are shown for an EHEC lysate sample and a mixture of five M_r_ protein standards, the latter of which pertains to the line with a low void volume peak at 667 kDa. Protein separations occurred in the 500-10 kDa range. On the right, protein bands visualized by Coomassie Blue staining in a 4–12%T SDS-PAGE gel are shown in the same order as the SEC fractions (with F3-s before pooling in two consecutive SEC fractions).

A schematic of the experimental strategy, which we term SEC-2D-LC-MS/MS here, is shown in ***Figure 1***. *E. coli* O157:H7 strain 86-24 cells were lysed using a lysozyme/EDTA spheroplasting method in the presence of 0.05% Triton X-100, followed by subsequent protein extraction of the pellet with a high salt solution (2.5 M NaBr). The combined solubilized fraction was separated by SEC yielding fractions in the M_r_ ranges >280 kDa, 280-80 kDa and 80-10 kDa, with estimated M_r_s based on retention times for five globular protein standards. As depicted in ***Figure 2***, the separation of proteins by SEC was also evident after denaturation and analysis in SDS-PAGE gels. These fractions, termed F1-s_SEC_, F2-s_SEC_ and F3-s_SEC_ (highest to lowest M_r_), and the insoluble lysate pellet (F4-p) were subjected to tryptic digestion. Four fractionations of tryptic peptide mixtures via SCX resulted in 13 samples each, which were sequentially analyzed by nano-flow C_18_-LC-MS/MS. This workflow was applied to 19 experiments yielding on average 270,000 mass spectra, with 35.2% of the mass spectra assigned to peptides predicted from the *E. coli* O157:H7 strain EDL933 protein sequence database. Using the Mascot algorithm, the average peptide FDR was 1.3%. For APEX-based protein quantification, Mascot-analyzed datasets or subsets of data derived from a distinct lysate fraction were rescored with Peptide/Protein Prophet, and quantities of proteins computed with a normalization factor based on ∼2.5×10^6^ proteins per *E. coli* cell [45].

### Protein quantification reproducibility

Eleven of the 19 SEC-2D-LC-MS/MS datasets were derived from extracts of bacterial cells grown in LB media to an OD_600_ of ∼2.0 and used for quantitative reproducibility assessments. Three stages of replication were considered, including the cell lysis stage (biological replicates, BR), the SEC separation stage (technical replicates, T_1_R) and the LC-MS/MS stage (technical replicates, T_4_R), as depicted in ***Figure 1***. Applying the Pearson correlation metric to APEX_i_ protein quantities of two datasets, the average R^2^ values were 0.71 (STDEV 0.05), 0.73 (STDEV 0.06) and 0.78 (STDEV 0.05) for the BR, T_1_R and T_4_R stages, respectively (n≥5 in each case). That the R^2^ values increased in this order was not surprising, since biological variation (at the BR stage) and more sample handling (at the T_1_R vs. the T_4_R stage) are expected to negatively impact reproducibility. The total number of proteins identified ranged from 1115 to 1861, averaging 1634 (STDEV 0.13). Limiting the analysis to proteins assigned to a specific subcellular or membrane localization using the PSORTb tool, the average R^2^ values were 0.79 (β-barrel outer membrane group, 47 proteins; STDEV 0.05), 0.79 (lipid-anchored group, 72 proteins; STDEV 0.02), 0.82 (soluble periplasmic group, 106 proteins; STDEV 0.05), 0.57 (group with two or more cytoplasmic TMDs, 236 proteins; STDEV 0.07) and 0.75 (soluble cytoplasmic group, 1308 proteins; STDEV 0.04). These calculations pertain to the T_1_R stage of replication (n = 5). Quantitative reproducibility levels for β-barrel OM and lipid-anchored proteins were relatively high, suggesting that these proteins were as effectively solubilized and digested as soluble periplasmic and cytoplasmic proteins. Proteins with two or more TMDs were less reproducibly quantified, clearly due to lower sequence coverage that results from lower abundance, lower solubility and less effective digestion in highly hydrophobic regions. The digestion method (FASP) was previously described as introducing little quantitative bias against hydrophobic membrane proteins [40] and was largely confirmed by our data. Non-ionic detergents (Triton X-100) and reagents with chaotropic properties (EDTA, 2.5 M NaBr) enhanced membrane protein solubilization [47], [48], [49]. Details of the R^2^ analyses are presented in ***Table S1***. R^2^ values for our entire dataset were lower compared to 2D-LC-MS/MS without the upfront SEC separation step [50], where the corresponding digestion, SCX and C_18_LC steps in addition to similar LTQ analysis parameters and sampling depth were applied. Those 2D-LC-MS/MS data resulted in R^2^ values of 0.84–0.88 at the T_4_R stage. Inserting more fractionation steps apparently decreased the repeatability of protein quantification. Repeatability and reproducibility of label-free shotgun proteomics are recognized as important criteria to assess data quality, particularly in biomarker discovery studies when quantitative accuracy is critical [21]. Further method improvements of the SEC-2D-LC-MS/MS workflow are desirable to reduce variability in protein quantification.

### Comparing *in silico* predicted and experimental *E. coli* O157:H7 protein profiles

Nine distinct pathovars have been recognized in *E. coli*, EHEC and EPEC (enteropathogenic *E. coli*) being the main pathovars that contain LEE in the genome and cause attachment and effacement (A/E) lesions in the colonic epithelium [36]. O157:H7 is the most prominent serotype associated with outbreaks of bloody diarrhea and severe complications including HUS, two of which were linked to strains 86-24 and EDL933, respectively [5], [51]. The strain 86-24, a producer of Stx2, was shown to be more virulent than EDL933, a producer of Stx1 and Stx2, in the gnotobiotic piglet model where oral infection was followed by severe neurologic injuries [9]. We used the strain 86-24 whose genome has not been sequenced for proteomic analyses of *in vitro* and piglet intestine-derived bacterial samples. The obvious choice of a strain whose genome has been sequenced for MS database searches was EDL933. Its genome is well characterized [2], the 86-24 and EDL933 strain differences in virulence in the piglet model seemed to be primarily linked to the Shiga toxin expression profile [9], both strains caused significant disease outbreaks in humans and are associated with the same MLST type st23, assessing the conservation of 15 distinct housekeeping gene sequences [52]. Finally, gene expression profiles of the two strains were recently compared, and substantial sequence similarities in several variable prophage regions can be inferred from successful DNA microarray analyses [53].

The subunit Stx2A of Shiga toxin 2 was identified in only one SEC-2D-LC-MS/MS experiment. Temporally repressed toxin expression, as reported for STEC-associated edema disease [54], and rapid secretion of the toxin, which limits intracellular Stx2 concentrations, are reasons that could account for low Stx2 abundance observed in the 86-24 proteome. The pathogenicity locus LEE encodes virulence factors contributing to A/E lesions upon contact with intestinal epithelial cells [36]. Twenty-one LEE-encoded proteins were identified in this survey, some with relatively high abundances including the outer membrane adhesion protein intimin (Eae), T3SS subunits (Esc proteins), translocator pore-forming Esp proteins, effector proteins (Tir and EspF), as well as proteins with chaperone (CesT) and regulatory functions (Ler, SepL). EHEC strains have an interesting repertoire of putative T3SS effectors encoded by genes outside of LEE [37]. Using the Sakai strain in the latter study, 39 proteins were experimentally confirmed to be secreted effectors, and the largest group was termed the NleG family. Fifteen of these effectors, half of them encoded by prophage regions in the EDL933 genome, were identified at low abundance levels in strain 86-24 lysates. The most reproducibly profiled of these effectors were NleG5-1 and NleG2-3. Interestingly, these and two other NleG family members were recently characterized as U-box E3 ubiquitin ligases with strong auto-ubiquitination activity *in vitro* [55]. This finding suggests that, upon injection into the epithelial host cell cytoplasm, these enzymes target the ubiquitin proteasome system, supporting the notion of molecular mimicry in order to perturb host cell functions. Generally low T3SS effector abundances surveyed here were not unexpected since secretion rapidly follows effector gene expression in the ribosome, and secreted fractions were not examined here. In a comparative analysis of wild-type strains of EHEC and EPEC, secretome profiles yielded higher abundance levels of several Esp effectors, whereas the respective Δ*ler* mutants, a key transcription factor inducing T3SS expression [56], did not. Indeed, two effectors that remain associated with the cell surface (Tir and Eae) were detected with higher abundance levels in the *E. coli* strain 86-24 proteome.

FliC, the antigenic determinant of the H antigen (H7 for strain 86-24) was reproducibly identified. Half of the 31 identified proteins encoded by genes of the virulence-associated plasmid pO157 were profiled with moderate to high abundances, including catalase/peroxidase (KatP), a putative α/β fold hydrolase (Ytp2) whose biological role is unknown and StcE. StcE is a metalloprotease that cleaves human C1 esterase inhibitor, mucin 7 and glycoprotein 340. The enzyme compromises human T-cell function and contributes to intimate adherence to the gut epithelium [57], [58]. It is a substrate of the pO157-encoded type 2 secretion system of which several subunits (EtpO, EtpG, EtpM and EtpE) were also identified in this survey. There are 16 and 19 prophage regions in the EDL933 and Sakai *E. coli* O157 genomes, respectively [59]. Searching the EDL933 database, we identified proteins in all but one of the 15 prophage regions and the infective phage BP-933W segment. However, only 16% of the phage-encoded proteins were identified from strain 86-24 lysates, and most of these low in abundance compared to 46.7% coverage for the entire EDL933 proteome. Low abundance and detection frequency are not necessarily signs of mutational decay, as a report on inducibility and release of *E. coli* O157 prophage DNA evidenced [59], [60]. In the LEE and CP-933T prophage segments, the proportion of identified proteins was relatively high (37% and 24%, respectively). It was lowest in the CP-933N prophage region (6.6%). Shotgun proteomic analysis of the *E. coli* K12 revealed that, while horizontally transferred genes mapping to the (phage) K-loops were as effectively transcribed as ‘core’ genes, proteins were expressed at a low percentage of ∼10% [61], which corresponds well with our data.

With a coverage of 46.7% (2521 of the 5397 predicted *E. coli* EDL933 proteins identified at a 1% FDR), this is the most comprehensive *E. coli* O157:H7 proteome analysis to date. Despite impressive technology advances, reports in which bacterial proteomes are profiled at a percentage above 50% of the genome-derived *in silico* proteome are rare. Among the reasons are: (1) silent genes, annotated as ORFs but not translated into proteins; (2) non-constitutively expressed genes repressed under most physiological conditions; (3) low abundance of proteins missed due to the stochastic peptide sampling mode in shotgun proteomics; (4) short polypeptides that are expressed but yield few or no proteotypic peptides. ***Table S2*** provides the entire list of proteins identified here with functional, structural, localization, physicochemical and APEX_i_ quantification data. A 2^nd^ worksheet (***Table S2***) pertains to use of a combination of two algorithms, InsPecT and MS-GF, which resulted in 1966 protein identifications at a 0.2% peptide FDR. ***Table S3*** contains the peptide dataset, also published in its entirety in the data repository PRIDE (http://www.ebi.ac.uk/pride/, Acc. No. 17108-17114). We are not reporting on differentially abundant proteins comparing the two physiological environments from which the STEC cells were isolated. These differences were extensive, influencing the activity of numerous biochemical pathways. Focusing here on the overall proteome and methodological improvements described in the following sections, we will cover proteomic adaptations of STEC to the pig intestinal environment in a separate publication.

### Fate of *E. coli* O157:H7 membrane proteins during cell lysate fractionation

Proteins localized in the inner membrane (IM) and OM were profiled comprehensively, with 458 of 1163 predicted integral IM proteins (39.4%) and 54 of 128 predicted OM proteins (42.2%). *In silico* predictions were based on subcellular assignments by the PSORTb tool. Lysis and solubilization reagents used here (Triton X-100, EDTA, lysozyme, 2.5 M NaBr) and tryptic digestions under denaturing conditions were sufficient to generate soluble peptides from many hydrophobic membrane proteins. Previously, expression of only 680 bitopic and polytopic IM proteins has been experimentally confirmed in 14 *E. coli* proteomic studies [62]. Low abundance membrane proteins were quantified less reproducibly which is a general concern of shotgun proteomics [21]. Membrane proteins were markedly enriched in fraction F1-s_SEC_ compared to the other three fractions, likely caused by high M_r_ mixed micelle formation with the detergent Triton X-100 and elution near the SEC void volume (∼500 kDa). Nearly 65% and 90% of all integral IM and OM proteins, respectively, were identified in fraction F1-s_SEC_. Given this observation, our data support the localization of orphan proteins such as YhcB, YjdB, YijP, YagU and YdgA, previously assigned to cell envelope protein complexes [63], in the IM. Examining APEX_i_ quantities of membrane proteins thought to be present in distinct stoichiometric ratios, we note that such data seem to be accurate in some, but less accurate in other cases. Succinate dehydrogenase (SdhABCD) is a tetrameric complex with a 1∶1∶1∶1 stoichiometry. SdhA and SdhB are peripherally bound to the membrane, while SdhC and SdhD have three TMDs in the IM. APEX_i_ quantities were 10156, 9057, 421 and 9943, respectively. SdhC was quantitatively underrepresented. The integral IM subunits of the cytochrome D terminal oxidase, CydA and CydB, were profiled in the expected 1∶1 ratio (APEX_i_ values of 3680 and 3220, respectively). The tetrameric ATP-binding cassette transporter Opp has two peripheral (OppD and OppF) and two integral (OppB and OppC) IM protein subunits, which are expected to have a 1∶1∶1∶1 stoichiometric ratio. The APEX_i_ quantities were 484, 343, 102 and 114, respectively, with both integral membrane subunits 3- to 4-fold less abundant. Interestingly, the soluble subunit OppA had a much higher APEX_i_ score (7116). This protein docks onto permease subunits (OppB/C) at the periplasmic surface to deliver oligopeptides for cytoplasmic import. While the observed quantitative ratios for subunits of membrane protein complexes need to be interpreted cautiously, the approach offers an opportunity to gain preliminary insights into protein complex composition. Data in ***Tables S2 and S4*** can be parsed to assess stoichiometric ratios of known protein complexes and, sorting by gene loci, potentially novel complexes formed by protein neighbors.

***Table S4*** lists APEX_i_ abundance values for proteins separately in the four surveyed fractions. Abundant β-barrel OM proteins such as OmpA, OmpC, OmpX, YciD and OmpT were retained to a higher extent in fraction F4-p than integral IM and lipoproteins, which is in agreement with studies on *E. coli* and *Campylobacter* membrane extracts where treatment of cells with Triton X-100 and EDTA resulted only in partial OM protein solubilization [49], [64]. Less hydrophobic β-barrel OM proteins appear to form mixed micelles with Triton X-100 less effectively. Peripheral membrane proteins were often profiled across the spectrum of SEC fractions, indicative of solubilization in the absence of Triton X-100: for example, subunits SdhA and SdhB, also detected as an *E. coli* succinate dehydrogenase sub-complex by blue native PAGE [65], subunits of ATP synthase (AtpH, AtpA and AtpD) and subunits of the main protein secretion system (SecA and SecB) were quite abundant in fractions F2-s_SEC_ and F3-s_SEC_, as opposed to the corresponding integral IM subunits of the complexes.

More than 50 uncharacterized low M_r_ proteins devoid of any predicted TMD or β-barrel motifs were enriched in fraction F1-s_SEC_. Due to the membrane protein enrichment in this fraction, we searched for the presence of N-terminal lipid anchor motifs associated with conserved signal peptidase II (Sp II) sequences. This analysis gave supporting evidence for lipid attachment of 38 proteins with the N-terminal Sp II motifs and 17 proteins with either less conserved motifs or unusual distances of the motif form the N-terminus not identified as lipoproteins by the algorithm LipoP. The proteins with an APEX_i_ score greater than 50 are listed in ***Table 1***. In some cases, we suspect incorrect prediction of the translational start site in the protein annotation of strain EDL933 as the reason for the failure to recognize the motif. Examples of proteins with atypical lipid anchor motifs are Z1474 (motif ITGC_17_A), YijI (motif CASC_49_S), Z0955 (motif FTTC_18_S), Z3360 (motif MKAC_44_S) and Z0984 (motif LCGC_27_G). Further support of membrane attachment was obtained for twelve of the 55 proteins via LC-MS/MS analysis of an iodixanol density gradient fraction highly enriched in bacterial membranes (see ***Table S5***). Data for all the 82 identified and assigned lipoproteins, including those previously characterized, are provided in ***Table S6***. Our approach did not allow specifying either IM or OM attachment of the proteins. Relying on transcript detection and lipoprotein motif searches, a study on *E. coli* strain MG1655 reported a roughly equal number of lipoproteins with unknown functions [66]. To our knowledge, lipoproteins of pathogenic *E. coli* strains have not been globally surveyed to date. Some of the lipoproteins, such as those encoded by prophage regions and absent in *E. coli* MG1655, may have unique functional roles in *E. coli* O157:H7. Unlike these lipid-anchored periplasmic proteins, soluble periplasmic proteins were almost invariably enriched in fraction F3-s_SEC_. The exceptions were proteins reported to interact with the OM: TolB and YbgF, which interact with the cell envelope complex Tol-Pal [67], and AsmA, which has been associated with the OM protein assembly complex [68].

**Table 1:** Putative EHEC lipoproteins markedly enriched in size exclusion chromatography fraction F1-s_SEC_.

Ca. A	Protein description B	Locus tag B	Gene name	Mr (Da)	Sub. Localiz. MPC C	PSort-B D	APEX F1–13 E	APEX F14–26 E	Sp II site F	Notes G
1	OM lipoprotein SlyB	Z2655	slyB	15601	L-OM	OM	31547	2607	LVGC_18_V	
3	uncharacterized protein YjeI	Z5749	yjeI	13064	L (hydr LS)	unkn	20511	3999	MAGC_30_S	
1*	entericidin B membrane lipoprotein	Z5754	ecnB	4809	L-IM, MPC-2	IM	11329	1749	LTAC_22_N	
2*	regulator in colanic acid synthesis	Z0208	rcsF	14177	L-OM	OM	8780	1375	LSGC_16_S	
2*	predicted lipoprotein YajG	Z0537	yajG	20977	L (IM or OM)	unkn	8499	520	LAGC_18_A	Δseq, 34
1	acridine efflux pump lipoprotein AcrA	Z0578	acrA	42195	L-IM, MPC-3	IM	8012	709	LTGC_25_D	
3	putative Bor protein of prophage CP-933X	Z1878	(borD)	10416	L (hydr LS)	unkn	7222	0	ITGC_17_A	s. Z1474
2*	protein DcrB involved in bacteriophage adsorption	Z4846	(dcrB)	19787	L (IM or OM)	PP	7205	505	LAAC_20_D	Δseq, 18
2*	putative lipoprotein YbjP	Z1095	ybjP	19150	L (IM or OM)	unkn	7180	37	LSAC_21_T	
2	putative lipoprotein YgdI	Z4126	(ygdI)	8342	L (IM or OM)	unkn	5912	1112	LSAC_21_S	
3	putative Bor protein of bacteriophage BP-933W	Z1474	(borW)	10477	L (hydr LS)	unkn	5666	0	ITGC_17_A	s. Z1878
2	OM starvation lipoprotein Slp	Z4908	slp	22210	L-OM	OM	5566	1211	LAAC_19_S	Δseq, 11
3	uncharacterized protein YijI	Z5503	yijI	16572	L (no hydr LS)	unkn	5532	4210	CASC_49_S	
2	uncharacterized lipoprotein YifI	Z5325	(yifI)	7177	L (IM or OM)	unkn	5265	569	LTGC_20_G	
2	put. collagen-binding surface adhesin protein YcfM	Z1744	ycfM	22515	L-OM	unkn	5166	0	LAGC_20_V	
2	predicted lipoprotein YedD	Z3018	yedD	15001	L (IM or OM)	unkn	4965	346	LAGC_16_A	
2*	predicted lipoprotein YraP	Z4509	yraP	20027	L (IM or OM)	PP	4549	392	LQGC_19_V	
2*	glycoprotein/polysaccharide metabolism protein	Z0565	ybaY	19440	L (IM or OM)	PP	3878	91	LAAC_19_A	
2	uncharacterized lipoprotein YgdR	Z4151	(ygdR)	7877	L (IM or OM)	unkn	3742	376	VSGC_19_S	
2	putative OM lipoprotein YiaD	Z4977	yiaD	22196	L (IM or OM)	OM	3322	45*	VSGC_21_T	
2*	conserved putative lipoprotein YiaF	Z4979	yiaF	30157	L (IM or OM)	unkn	3297	157	LSGC_26_F	
2	predicted metallopeptidase YggG	Z4280	yggG	31826	L (IM or OM)	unkn	2523	326	LTGC_19_Q	Δseq, 42
2	putative glycosylase/lipoprotein YraM	Z4506	yraM	72808	L (IM or OM)	IM	2431	145	FVGC_27_G	
2	put. c-type lysozyme inhibitor lipoprotein YdhA	Z2653	ydhA	9553	L (IM or OM)	unkn	2013	472	LTGC_18_S	Δseq, 27
2	OM lipoprotein/putative pectinesterase	Z0943	ybhC	45979	L-OM	OM	1830	91	LTAC_22_S	
2	predicted outer membrane lipoprotein YfeY	Z3697	(yfeY)	20814	L (IM or OM)	unkn	1775	0	LTGC_18_S	
3	unknown protein encoded by prophage CP-933K	Z0955	-	34904	L (hydr LS)	unkn	1564	0	FTTC_18_S	
2	predicted exopolysaccharide export protein GfcE	Z1400	gfcE	41739	L (IM or OM)	OM	1386	43	LTAC_21_T	
2*	putative OM lipid asymmetry protein MlaA (VacJ)	Z3610	mlaA	28007	L (IM or OM)	OM	1300	0	LVGC_18_A	
2*	putative OM-associated lipoprotein	Z2849	(yeaY)	20866	L (IM or OM)	OM	1040	0	LSGC_23_V	
2	uncharacterized lipoprotein YdcL	Z2287	ydcL	24426	L (IM or OM)	unkn	901	0	LSGC_21_A	
2	type II secretion protein	L7044	etpO	15051	L (IM or OM)	mscl	901	36	ISGC_24_H	
3	unknown protein encoded by prophage CP-933V	Z3360	-	19772	L (no hydr LS)	unkn	858	0	MKAC_44_S	
2	conserved membrane lipoprotein YoaF	Z2835	yoaF	8899	L (IM or OM)	unkn	853	0	LAGC_17_S	
2	predicted lipoprotein YceB	Z1700	yceB	20469	L (IM or OM)	unkn	833	67	LVGC_19_N	
3*	unknown protein encoded by prophage CP-933K	Z0984	-	11416	L (no hydr LS)	unkn	563	0	LCGC_27_G	s. Z2148
3	heat-inducible protein HslJ	Z2330	hslJ	15181	L (IM or OM)	unkn	350	29	MAGC_17_V	
2	uncharacterized protein L7092	L7092	-	16108	L (IM or OM)	unkn	321	0	VAGC_31_A	
3	uncharacterized protein YegR	Z3251	(yegR)	13836	L (IM or OM)	unkn	276	37	MSGC_38_A	
2	putative α-macroglobulin	Z3787	(yfhM)	181409	L (IM or OM)	mscl	261	4*	LAGC_18_D	
3	uncharacterized protein YdjY	Z2783	ydjY	30511	L (hydr LS)	PP	207	0	LSGC_81_D	Δseq, 54
2	uncharacterized lipoprotein YajI	Z0513	yajI	19602	L (IM or OM)	unkn	169	0	LSAC_40_V	Δseq, 20
2*	putative tetratricopeptide repeat lipoprotein	Z4203	(ygeR)	23480	L (IM or OM)	unkn	130	24	LAGC_13_S	Δseq, 13
2*	put. G4C polysaccharide synthesis lipoprotein YmcA	Z1401	ymcA	78668	L (IM or OM)	OM	120	9	SSAC_19_H	
2	hypothetical protein Z4394	Z4394	ygiB	23478	L (IM or OM)	PP	110	46	LAGC_37_E	
2	putative lipoprotein YnbE	Z2327	ynbE	6815	L (IM or OM)	unkn	109	0	LVGC_17_T	
2	conserved lipoprotein YmbA	Z1302	ymbA	19949	L (IM or OM)	unkn	107	0	LAGC_11_S	
2	predicted outer membrane lipoprotein YfgH	Z3769	yfgH	17704	L (IM or OM)	OM	97	0	LAGC_22_Q	
3	put. cell division inhibitor (prophage CP-933U)	Z3128	-	6991	L (no hydr LS)	unkn	94	0	SEGC_14_F	
2	conserved putative lipoprotein YfhG	Z3831	yfhG	27347	L (IM or OM)	unkn	78	0	LLGC_26_V	
2	put. G4C polysaccharide synthesis lipoprotein YmcC	Z1403	ymcC	24248	L (IM or OM)	IM	61	0	LAGC_16_T	
2	predicted thiamine biosynthesis lipoprotein ApbE	Z3472	yojL	38547	L (IM or OM)	unkn	52	0	FVGC_25_D	
2	uncharacterized protein YaiW	Z0474	yaiW	40416	L (IM or OM)	unkn	52	0	LAGC_21_S	

***A***: Categories. 1, verified lipoprotein (three listed out of >20); 2, conserved Sp II signal peptidase motif with a hydrophobic leader sequence; 3, tentative Sp II signal peptidase motif; *protein was also identified in a membrane phospholipid-enriched cell lysate fraction derived from iodixanol density gradient centrifugation experiments.

***B***: Protein annotations from the databases EcoCyc or *E. coli* O157:H7 strain EDL933; gene name (in parentheses): listed only in the UniProt.

***C***: Prediction of subcellular localization and other annotation features such as multi-protein complex (MPC) with 2 or more subunits based database entries; L: lipoprotein; IM, inner membrane; OM, outer membrane; no hydr/hydr LS (presence/absence of hydrophobic leader sequence N-terminal to Sp II motif).

***D***: Subcellular localization prediction based on PSORTb assignment; unkn (unknown); PP (periplasm); CY (cytoplasm); mscl (multiple localizations).

***E***: Average APEX_i_ protein abundance score (n = 12) for protein in size exclusion chromatography fractions F1-s_SEC_ (F1–13, Mr >280 kDa) where membrane proteins were enriched and F2-s_SEC_ (F14–26, Mr 280-80 kDa); *score from fraction F3-s_SEC_ if score was ‘0’ in F2-s_SEC_.

***F***: Signal peptidase II motif surrounding the lipid-anchored cysteine residue; tentative motif assignment for category 3 proteins. The number denotes the Cys amino acid position in the protein sequence provided in the *E. coli* O157:H7 EDL933 database.

***G***: Δseq: likely incorrect prediction of translational start site in *E. coli* O157:H7 EDL933 database (number of amino acids represents the respective shift based on our corrections); s. Z2148, Z1474 etc.: high sequence similarity among genes encoded by prophage regions.

### Fractionating soluble proteins associated with higher order complexes

Abundance comparisons of proteins predicted to be soluble, either cytoplasmic or periplasmic, across the SEC fractions F1-s_SEC_, F2-s_SEC_ and F3-s_SEC_ were used to assess their enrichment in M_r_ ranges discordant with that of monomeric forms. Incorporating information on homooligomeric and heterooligomeric assemblies for many *E. coli* proteins, we were able to compare the experimentally determined M_r_ intervals for protein elution via SEC with protein sequenced-derived monomeric and native M_r_ values described for protein complexes in *E. coli* databases. EcoCyc and the *E. coli* data subset in Uniprot were queried to extract annotated native M_r_ values for more than 500 proteins part of complexes. As described in detail in ***Table S4***, the data revealed excellent agreements, but also differences between our experimental M_r_ estimates and those reported in the databases: a rank matrix for APEX_i_ quantities of each protein observed in a given fraction was established to facilitate comparing the SEC-derived ‘protein peaks’ with reported oligomeric M_r_s. It is noteworthy mentioning a few confounding factors: (1) protein complexes fall apart upon cell lysis and solubilization in a largely unpredictable manner; (2) SEC permits only estimates of true M_r_ values of protein complexes and tends to be less accurate if non-globular shaped proteins are involved [69]; (3) our methodology was unable to reveal specific protein interaction partners when a M_r_ value for a protein suggested a complex.

The comparisons revealed stronger experimental evidence for intact protein homooligomers than for large heterooligomeric protein complexes. For example, the pyruvate dehydrogenase multienzyme complex consists of 60 subunits: AceE_24_AceF_24_Lpd_12_ (the subscript number indicates the number of subunits in a given complex). All three subunits were profiled over the entire M_r_ range (SEC void volume to 10 kDa). Proteins enriched in a SEC fraction concordant with the M_r_ estimate of known complexes were: Ftn_24_, Kbl_12_, KatG_4_, GlpK_4_, GltA_4_, FbaB_10_, MaeB_6_, RpoABCDE and GlnA_12_ (fraction F1-s_SEC_); Udp_6_, IcdA_2_, TnaA_4_, GapA_4_, GlpQ_2_, Fba_2_, AcnB_2_, TktA_2_, AspC_2_, Gnd_2_ and Ppa_6_ (fraction F2-s_SEC_). Complexes that were largely disintegrated were RibH_60_, ClpB_4_, ClpA_12_ClpP_14_, Lpd_2_GcvP_2_GcvH_1_GcvT_1_, HtrA_6_ and Dps_12_. Having verified intact complexes, all profiled proteins not characterized as subunits of higher order assemblies to date were examined, resulting in ∼190 proteins for which participation in a complex is now predicted. We required the monomeric M_r_ value to be least 25% below that of the lower assigned M_r_ limit in SEC experiments (80 and 280 kDa for F1-s_SEC_ and F2-s_SEC_, respectively) to assign a new complex-associated protein. The two categories of proteins, those reported vs. not reported to participate in protein complexes, are distinguished by their color codes in ***Table S4***.

**Figure 3:**
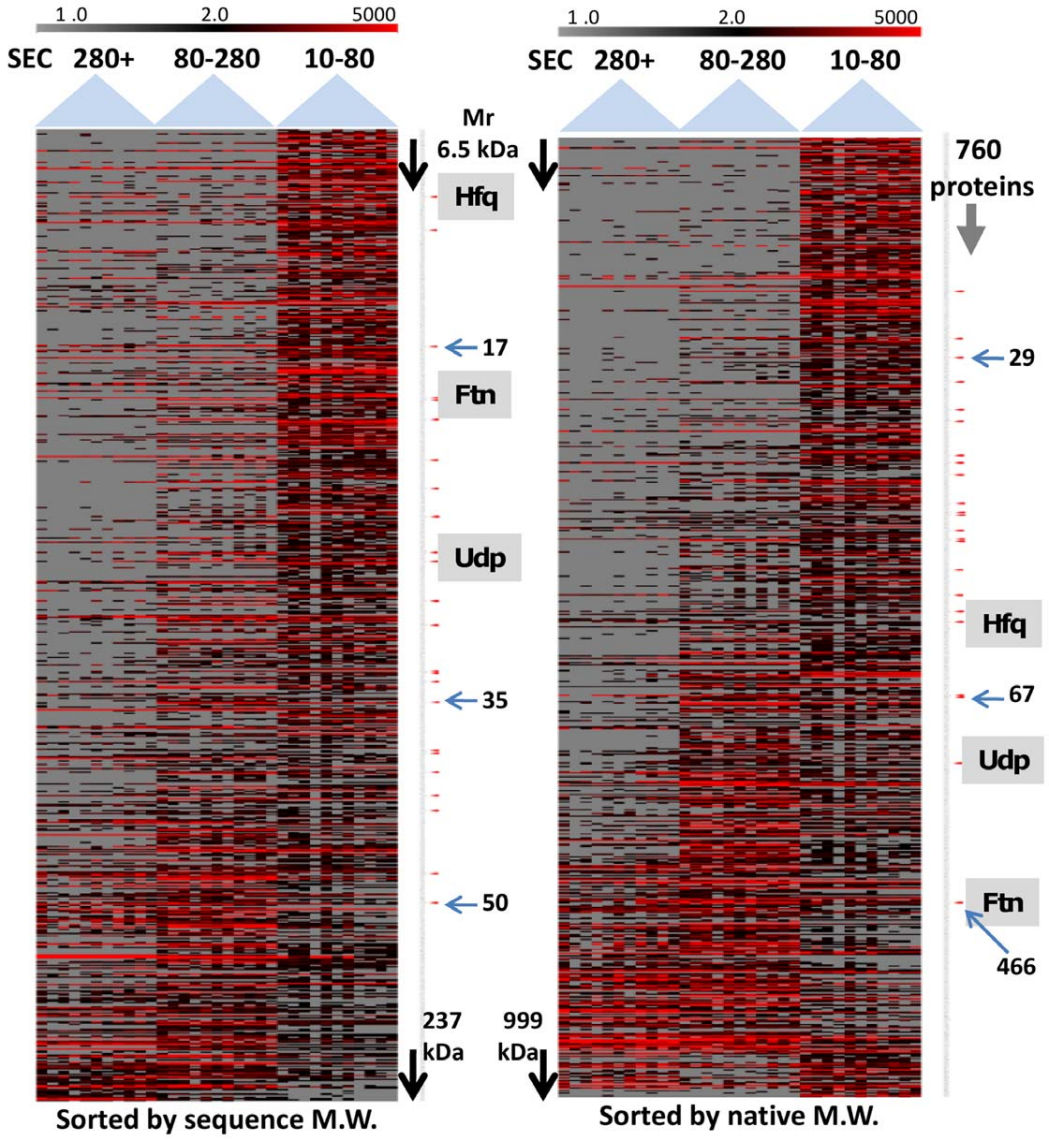
Correlation of native and sequence-based M_r_ values of proteins with their quantitative elution profiles in SEC fractions associated with three approximate M_r_ ranges (F1-s_SEC_, >280 kDa, F2-s_SEC_ 280-80 kDa, F3-s_SEC_ 80-10 kDa). Each heat map column under the broad blue arrow indicating a M_r_ range represents 11 individual, replicate APEX_i_ datasets. Each row corresponds to one of 760 EHEC proteins, quantified with the APEX_i_ tool, that were altered with statistical significance using the Kruskal-Wallis test (p-value<0.02) comparing protein abundances in the three SEC fractions (F1-s_SEC_, F2-s_SEC_ and F3-s_SEC_). The range of colors in the heat map corresponds to APEX_i_ values from 0 to 5000. Left heap map: proteins are ordered based on calculated, amino acid sequence-based M_r_ values; right heat map: proteins are ordered based on native M_r_ values including homooligomers and multi-protein complexes (for calculations, see *Table S7*). The maximum M_r_ value was 999 kDa, including larger complexes such as the ribosome. Protein positions are indicated for the monomer/complex as follows: Ftn/Ftn_24_, Udp/Udp_6_ and Hfq/Hfq_6_. The correlation of protein elution patterns by SEC with calculated native M_r_s is improved.

We took this analysis a step further, classifying soluble proteins that were enriched with statistical significance in a distinct SEC fraction. Proteins with predicted/experimental evidence for membrane integration or attachment were excluded from the analysis. A Kruskal-Wallis test returned 760 proteins as significant with a p-value set at <0.02 in a three-group analysis (F1-s_SEC_ vs. F2-s_SEC_ vs. F3-s_SEC_). To visualize how the proteins were quantitatively distributed among the fractions (with analytical replicates) the data were displayed in a heat map, ordered either based on monomeric or native (protein complex) M_r_ values (***Figure 3***). While the patterns were noisy, in part due to numerous proteins for which native M_r_ data are unavailable, the expected M_r_ distribution was more evident in the heat map ordered based on native protein M_r_ values. The exception was proteins at the very bottom of the heat maps, many of which were assigned a maximum M_r_ of 999 kDa in the pattern analysis. A lower level of correlation with such high native M_r_ values was likely caused by disintegration of large complexes, e.g. the ribosome whose subunits were the majority of proteins in this part of the graphic. Ribosomal proteins were most abundant in fraction F3-s_SEC_. The reagent EDTA, added to the lysis buffer, lowers free Mg^2+^ which probably destabilized the ribosome and led to the release of disassembled low M_r_ subunits [70]. The dataset pertaining to these heat maps is provided in ***Table S7***. Then we performed a Pearson correlation-based hierarchical clustering analysis to assess whether many of the 190 predicted, complex-associated proteins clustered with proteins part of established complexes in *E. coli*. This was indeed observed, as shown for a selected list of proteins in ***Table 2*** and in a graphic revealing protein clusters in ***Figure S1***. The clusters C1 and C2 featured protein enrichment in fraction F1-s_SEC_ (like Ftn_24_); the cluster C3 represented proteins most abundant in fraction F2-s_SEC_ (like Udp_6_). For several proteins associated here with higher order assemblies (including some in ***Table 2***), protein-protein interactions have been suggested. The *E. coli* interactome published by Hu *et al.* [32] reported interactions of the proteins PrsA, YbeZ, YeeX, YggE and YfbU with ribosome subunits, of YbeZ with RraA and of YfbU with YeeX. PrsA localized in a small cluster with a majority of proteins being ribosomal. YbiB and YbiC, each a protein of unknown function, and YlbA and YlbC, each believed to contribute to allantoin assimilation, are encoded by adjacent genes whose products may be subunits of a complex. Only a few proteins encoded by prophage loci or plasmid pO157 were identified as protein candidates for oligomeric assemblies: catalase KatP and the uncharacterized proteins L7060 (plasmid pO157) and Z2386 (CP933-R region). In summary, this data lends further support to the notion that our methodology to associate uncharacterized proteins with complex formation is biologically meaningful.

**Table 2:** Selection of proteins predicted to participate in di- or oligomeric complexes based on HCL analysis of SEC fraction abundance patterns.

Cl. *A*	Protein descriptions *B*	Locu tag *B*	Gene name	M.W. *(kDa)*	Sub. Localiz. MPC *C*	KWS *p-value D*	APEX_Av_ *>280 E*	APEX_AV_ *280-80*	APEX_AV_ *80-10*	Functional roles *F*
C1	RNA-binding protein Hfq	Z5779	hfq	11166	CY, Ho-6	1.95E-02	25095	9957	2079	mRNA degradosome component
C1	putative acetoin dehydrogenase UcpA	Z3691	ucpA	27831	CY	2.73E-03	4843	5038	1953	(oxidoreductive) sulfate metabolism
C1	short chain dehydrogenase YbbO	Z0646	ybbO	29428	CY	1.95E-02	377	253	0	oxidoreductive processes
C2	conserved protein YicC	Z5069	yicC	33158	CY	1.39E-04	2382	635	0	unknown function
C2	ferritin	Z2960	ftn	19423	CY, Ho-24	3.11E-05	56748	14396	7016	iron transport and binding protein
C2	putative ATP-bdg. protein in pho regulon	Z0809	ybeZ	40653	CY, RIB-ppi	1.95E-05	5273	749	356	unknown function
C2	phosphoglucosamine mutase	Z4538	glmM	47545	CY	2.07E-06	4799	2061	117	UDP-NAc-glucosamine biosynthesis
C3	molybdenum cofactor biosynth. protein	Z0009	mogA	21222	CY, Ho-3	9.39E-07	0	1939	81	cofactor biosynthesis
C3	D-lactate dehydrogenase	Z2329	ldhA	36519	CY	1.93E-05	92	2477	109	mixed acid fermentation
C3	ureidoglycolate dehydrogenase	Z0672	ylbC	37889	CY	7.88E-04	0	666	43	allantoin assimilation
C3	D-glucuronate/galacturonate isomerase	Z4445	uxaC	53927	CY	4.60E-05	0	466	0	carbohydrate metabolic process
C3	oxidative stress response protein	Z4259	yggE	26634	PP, RIB-ppi	9.62E-06	1541	5964	31	increased by AI-2 quorum signaling
C3	agmatinase	Z4281	speB	33556	CY, Ho-2	4.24E-06	852	5326	0	2nd step in putrescine biosynthesis
C3	succinylglutamic semialdehyde dehydrogenase	Z2778	astD	52983	CY	1.35E-06	0	2849	86	arginine catabolic pathway
C3	predicted transferase/phosphorylase	Z1021	ybiB	35183	CY	2.01E-06	0	2998	251	unknown function
C3	uridine phosphorylase	Z5353	udp	27149	CY, Ho-6	1.58E-05	2893	23958	3090	purine and pyrimidine biosynthesis
C3	conserved protein YfbU	Z3555	yfbU	19536	CY, RIB-ppi	3.01E-06	43	13088	512	unknown function
C3	tartronate semialdehyde reductase	Z4477	garR	30658	CY	1.80E-06	102	4430	37	carbohydrate acid metabolism
C3	putative chromate reductase	Z5208	yieF	20375	CY, Ho-2	6.99E-07	0	13599	5096	flavoprotein containing FMN cofactor
C3	ribulose-phosphate 3-epimerase	Z4739	rpe	24553	CY	2.13E-06	233	4390	68	carbohydrate metabolism
C3	nucleoid-associated protein YejK	Z3445	yejK	37822	CY	1.63E-05	338	1890	21	unknown function
C3	glyoxylate/hydroxypyruvate reductase	Z1666	ghrA	36853	CY	1.91E-04	91	2819	541	response to cellular stress
C3	predicted dehydrogenase YbiC	Z1022	ybiC	38930	CY	1.33E-04	319	3683	477	unknown function
C3	ribonuclease activity regulator protein RraA	Z5476	rraA	17360	CY, ybeZ-ppi	5.29E-05	2331	7463	1899	mRNA degradosome component
C3	S-ureidoglycine aminohydrolase	Z0670	ylbA	28731	CY	5.34E-03	1930	4266	118	allantoin assimilation
C3	peptidyl-prolyl cis-trans isomerase	Z5818	fklB	22237	CY/PP, Ho-2	3.82E-07	0	11764	3295	protein folding in periplasm
C3	Xaa-Pro dipeptidase	Z5369	pepQ	50194	CY	6.19E-03	1411	4303	1069	peptide catabolic process

***A***: Clusters derived from Hierarchical clustering (HCL) using the Pearson correlation metric in the software MeV featuring groups of proteins with similar SEC fractionation patterns.

In each cluster, more than 70% of the proteins were subunits of known homo- or hetero-oligomeric protein complexes with M.W. values in better agreement with a complex than monomer. Examples of such characterized proteins are ferritin and uridine phosphorylase. Clusters are also visualized in Figure S2 (Suppl. Information).

***B***: Gene name, locus tag and protein description are derived from annotations in the *E. coli* O157:H7 EDL933 genome (UniProt database) or the EcoCyc database.

***C***: Subcellular localization derived from EcoCyc data or annotations in UniProt for the EDL933 genome and multi-protein complexes (MPC). CY: cytoplasm; PP, periplasm; Ho-‘2’, Ho-6’ etc., homo-oligomeric protein with the number of subunits; ppi, proven protein interactions with another protein or a large assembly such as the ribosome (RIB).

***D***: KWS: the Kruskal-Wallis statistic was applied to the entire EHEC proteome dataset (2521 proteins) to select proteins with statistically significant abundance differences comparing. the size exclusion chromatography (SEC) fractions (>280 kDa, 280-80 kDa and 80-10 kDa). After elimination of integral membrane proteins and setting the KWS p-value at <0.02, 760 proteins were selected for the HCL analysis.

***E***: Average protein abundances (APEX_i_ scores) in each of the three SEC fractions. n = 11; normalization factor: 2.5×10^6^; ‘0’: no peptide spectra were detected for a given protein.

***F***: Functional roles of proteins predicted for the EHEC protein or the respective *E. coli* ortholog (data from EcoCyc or UniProt annotations).

### Proteins enriched in insoluble lysate fractions and prone to aggregation

More than 1,000 proteins were routinely identified in LC-MS/MS analyses of the insoluble fraction (F4-p). We have already referred to the retention of membrane proteins - particularly those of the OM - in the insoluble *E. coli* O157:H7 pellet, likely caused by incomplete protein solubilization. F4-p protein profiles also revealed high prevalence of numerous proteins not associated with membranes, which was supported by statistical evidence using a Wilcoxon rank sum test that compared protein abundances in the three SEC fractions vs. the F4-p fraction (***Table S7***). At a p-value of <0.02, 800 proteins displayed a statistically significant pattern of enrichment in either F4-p or the combined SEC fractions. Given the considerable knowledge on insoluble protein aggregates formed in *E. coli* cells, our proteomic and literature data were mined to determine whether a pattern of protein characteristics evolved that linked specific types of proteins with aggregation/insolubility behavior. Data related to protein propensities to aggregate are included in ***Table S4*** (worksheet F4-p).

To determine that membrane proteins prevalent in fraction F4-p were indeed a fraction discernible from aggregates of intracellular proteins, the bacterial cell lysate was subjected to iodixanol density gradient centrifugation. Two opaque layers were isolated at a density of ρ = 1.21 g/cm^2^ (H_D_) and at a lower density of ρ = 1.13 g/cm^2^ (L_D_). LC-MS/MS analysis (***Table S5***) revealed that the L_D_ layer was enriched in membrane proteins including highly prevalent OM proteins, whereas the H_D_ layer was enriched in cytoplasmic proteins which apparently aggregated and were rendered insoluble after cell lysis. The overlap of proteins identified in the H_D_ gradient fraction with those of high abundance in F4-p fractions from the main analysis was extensive, further evidence in support of the assumption that F4-p was enriched in proteins forming insoluble aggregates. Soluble periplasmic proteins were underrepresented in F4-p and absent in the H_D_ fraction, supporting the notion that such proteins, which are subject to folding pathways in more oxidative environments [71], have a lower propensity to aggregate. Similar conclusions were drawn in a study on protein aggregation using healthy *E. coli* cells [27]. This is not to say that protein aggregation does not occur in the periplasm of *E. coli* and other enteric pathogens. Under acid stress conditions, two small acid stress response chaperones (HdeA and HdeB) act as a pH-optimized periplasmic protein disaggregation system [72] that is induced in *Shigella dysenteriae* in the mammalian gut environment [39]. Ninety% of the ∼120 *E. coli* proteins described as aggregate-forming thermolabile substrates of the chaperones DnaK, GroEL, ClpB and ClpX/P [28], [73] were also detected, and typically enriched, in the F4-p fraction in our survey.

Ribosomal protein subunits were markedly enriched in F4-p, tempting us to explore whether proteins participating in large organelle-like structures are generally prone to form aggregates, as observed for non-pathogenic *E. coli* aggregates [27]. A third of the 100 proteins of highest abundant in fraction F4-p were ribosomal or ribosome-associated elongation factors (***Table S4***). GlpD and GlpA/GlpB, two glycerol-3-phosphate dehydrogenases [74], [75], and glycerol kinase (GlpK) were very abundant in fraction F4-p. Interaction of GlpK, but not of the dehydrogenases, with the inner membrane glycerol facilitator protein was reported [76]. We speculate that a large transport-and-degradation complex mediating glycerol import and oxidation, involving these proteins, exists at the IM cytoplasmic surface.

We also noticed significant enrichment in F4-p and the H_D_ fractions of several enzymes contributing to O-antigen building block biosynthesis and assembly. Most of the enzymes are part of a large gene cluster encompassing the gene loci Z3189-Z3206. ***Table 3*** lists the 16 O-antigen biosynthesis proteins detected here and three additional enzymes associated with mobilization of galactose, an O-antigen sugar precursor. We hypothesize that the proteins co-localize at the cytoplasmic surface of the IM since some components required for biosynthesis of LPS, the outermost OM layer, are integrated in the IM, thus forming an ‘O-antigen biosynthesome’. Enzymes responsible for lipid A-core biosynthesis and encoded by the *waa* and *lpx* genes displayed less consistent enrichment in fraction F4-p. These enzymes represent the biosynthetic branch for synthesis of the LPS core molecule of *E. coli* O157 and have been linked to an integrated structure in the IM periphery [77]. To our knowledge, there is no other evidence for an integrated O-antigen biosynthesis apparatus. The precise pathway of the serotype O157 antigen biosynthesis remains to be elucidated, since several of the glycosyltransferase (WbdR, WbdQ, WbdP, WbdO and GalF) and the epimerase (Gne) activities have not been verified [78]. Purifying the predicted ‘O-antigen biosynthesome’ and protein-protein interaction screens will be useful to reveal structure-function relationships, with the potential of small molecule inhibitor design targeting one or several of the biosynthetic steps. Abrogation of O-antigen biosynthesis is an attractive pathway of interference with the pathogen's recognition by or evasion from the human immune system.

**Table 3:** Proteins predicted to participate in multi-protein structures such as cell division and O-antigen biosynthesis, and prone to aggregation based on relative abundance in insoluble *E. coli* O157:H7 fraction.

Cl. *A*	Protein Description *B*	Locus tag *B*	Gene name	M.W. (kDa)	Sub. Localiz. *C*	APEX_Av_ F1–39 *D*	APEX_AV_ F40–52	W-test p- value *E*
C3	aldose 1-epimerase	Z0926	galM	38176	CY	964	4334	4.14E-05
C1	galactokinase	Z0927	galK	41504	CY	15	3871	3.60E-10
C3	UTP-glucose-1-phosphate uridylyltransferase	Z2012	galU	32941	CY	1431	4089	1.37E-05
n.d.	regulator of LPS O-antigen chain	Z3189	wzzB	37810	IM	868	144	n.d.
C2	UDP-glucose 6-dehydrogenase	Z3190	ugd	43711	CY	0	1133	1.11E-09
n.d.	putative 6-phosphogluconate dehydrogenase	Z3191	gnd	51475	CY	6111	503	n.d.
n.d.	putative N-acetyl transferase	Z3192	wbdR	23742	CY	53	261	n.d.
C1	phosphomannomutase	Z3194	manB	50338	CY	91	4107	5.23E-08
C2	mannose-1-P guanosyltransferase	Z3195	manC	54268	CY	44	6027	1.06E-09
C3	GDP-mannose mannosylhydrolase (gmm)	Z3196	wbdQ	19567	CY	23	4400	1.06E-09
C3	fucose synthetase	Z3197	fcI	36267	CY	170	4328	4.75E-08
C3	GDP-mannose dehydratase	Z3198	gmd	41673	CY	557	8901	4.62E-07
C2	putative glycosyl transferase	Z3199	wbdP	46609	CY	94	2026	2.43E-08
C3	perosamine synthetase	Z3200	per	41553	CY	774	7924	6.71E-07
n.d.	putative glycosyl transferase	Z3202	wbdO	28358	CY	0	153	n.d.
o.C	putative glycosyl transferase	Z3204	wbdN	30345	CY	0	485	4.96E-05
C3	UTP-glucose-1-P uridylyltransferase subunit	Z3205	galF	32828	CY	199	6319	1.08E-07
C3	putative UDP-galactose 4-epimerase	Z3206	gne	37141	CY	209	5271	5.23E-08
o.C	predicted acetyltransferase	Z5427	yiid	37065	CY	0	851	1.04E-07
C2	cell division protein FtsA	Z0104	ftsA	45328	Z-ring, pIM	0	1498	1.11E-09
C3	cell division protein FtsZ	Z0105	ftsZ	40322	Z-ring, pIM	1562	5064	1.72E-05
C2	UDP-3-O-[3-hydroxymyristoyl] N-AcGln deacetylase	Z0106	lpxC	33955	CY	0	2432	9.87E-11
C2	cell division protein FtsE	Z4837	ftsE	24438	pIM	0	1995	1.13E-08
o.C	cell division factor ZapB	Z5473	zapB	9635	Z-ring, CY	9067	1087	2.72E-05
C3	cell division topological specificity factor	Z1936	minE	10235	CY, pIM	1781	4716	2.83E-03
C3	cell division inhibitor MinD	Z1937	minD	29613	pIM	180	10936	7.70E-08
C2	septum formation inhibitor MinC	Z1938	minC	24745	CY, pIM	110	1190	6.93E-07
C3	penicillin binding protein 3, MreB	Z4610	mreB	39019	CY, pIM	0	7334	9.87E-11
C1	plasmid-partitioning protein SopA	L7068	sopA	43658	CY	0	4258	9.87E-11
C1	plasmid-partitioning protein SopB	L7069	sopB	35371	CY	0	3232	9.87E-11
o.C	elongation factor Tu	Z4697	tuf	43282	CY, pIM	22954	88106	9.97E-07

***A***: Hierarchical clustering analysis using the Euclidian distance metric in MeV featuring groups of proteins with similar abundance patterns in the three SEC fractions compared to insoluble *E. coli* strain 86-24 lysate fraction. Clusters are visualized in Figure S2 (Suppl. Information). C1, C2 and C3 clusters contained many proteins part of large subcellular assemblies; o.C: other cluster; n.d. not significant in W-test.

***B***: Gene name, locus tag and protein description are derived from annotations in the *E. coli* O157:H7 EDL933 genome (UniProt database) or the EcoCyc database. The first 19 genes/proteins have been associated with biosynthesis of the ‘O157’ O-antigen of EHEC serotype O157:H7. The next 12 genes/proteins have been associated with the cytoskeleton and cell (or plasmid) division. The flippase Wzx and polymerase Wzy, also part of the O-antigen biosynthesis apparatus and rich in transmembrane domains were not observed.

***C***: Subcellular localization of proteins derived from EcoCyc data or annotations in UniProt for the EDL933 genome. CY: cytoplasm; IM: inner membrane; Z-ring: Z-ring formation during cell division septation; pIM: peripheral IM protein.

***D***: Average protein abundances (APEX_i_ scores) in the combined size exclusion chromatography (SEC) fractions (F1–39) pertaining to the soluble lysate fraction and the insoluble lysate fraction F4-p (F40–52). n = 11; APEX_i_ normalization factor: 2.5×10^6^; ‘0’: no peptide spectra were detected for a given protein.

***E***: W-test: the Wilcoxon Rank Sum test was applied to the entire EHEC proteome dataset of 2521 proteins to select proteins with statistically significant abundance differences comparing the combined SEC fractions (F1–39; >280 kDa, 280-80 kDa and 80-10 kDa) with the insoluble lysate fraction F4-p. The proteins with a p-value of <0.02 (802 entries) were used for Hierarchical Clustering using the Euclidian distance metric.

We also observed enrichment in F4-p of proteins with major roles in the biogenesis of the cytoskeleton of rod-shaped bacteria, including the bacterial actins MreB and FtsA [79], [80], the MreB-interacting elongation factor Tu [34], the bacterial tubulin FtsZ [80], [81], the cell division-regulating system MinCDE [80] and the polymer-forming SopA/SopB system that controls plasmid segregation during cell division [82]. With the exception of SopA/SopB, these structures have also been associated with the IM periphery. In addition to FtsA/FtsZ, the core structure of cell division septa, two other components of the cell division-initiating Z-ring (FtsE and YhdE) were enriched in F4-p. Most proteins associated with O-antigen biosynthesis or cytoskeletal elements were enriched in fraction F4-p with statistical significance (***Table 3***). In further support of the aggregation behavior of such large subcellular protein assemblies, many ribosomal, O-antigen biosynthesis and cytoskeletal proteins were identified in the density gradient fraction H_D_ (see Notes, ***Table S5***). Finally, most subunits part of these higher order protein assemblies also clustered in a Euclidian distance clustering analysis, which is depicted in ***Figure S2***.

Many DNA-binding subunits of two-component transcriptional regulators were also enriched in fraction F4-p (e.g. ArcA, PhoP, OmpR and RstA), as were enzyme complexes harboring iron-sulfur cofactors (e.g. AcnA, AcnB, FumA, SdaA and PflA/PflB). We examined whether other organelle-like assemblies in bacteria had enrichment patterns similar to those mentioned previously. Although individual proteins were enriched in fraction F4-p, this was not generally observed for the tRNA synthetase complex [83], the mRNA degadosome [35] and the purinosome. Purinosome (purine biosynthesis) subunits enriched in F4-p were Add, PurC, PurM and PurT. Unlike O-antigen biosynthesis and cell division, these structures are not known to be IM-associated. Among the uncharacterized proteins whose genes clustered at a specific locus, we noticed that YeaD, YeaG and YeaK (loci Z2820-27) were enriched in fraction F4-p, and speculate that these proteins form a functional unit in the *E. coli* O157:H7 cell.

### Concluding remarks

We developed and assessed the reproducibility of a shotgun proteomics approach incorporating a SEC step to separate proteins according to their M_r_ values prior to LC-MS/MS. The strategy was applied to a virulent *E. coli* O157:H7 strain, isolated from the large bowel of infected piglets and from cell cultures, and included quantification of more than 2500 proteins via computationally modified spectral counting. Only 13% of the ORFs encoded by phage/prophage regions were expressed at the protein level, mostly at low abundance. Defective prophages are not simply genetic remnants in the course of EHEC evolution but are inducible and released from cells as particulate DNA, disseminating virulence-related genes [60]. Coverage of proteins encoded by plasmid pO157 and the LEE pathogenicity island, each producing functional virulence factors, was markedly higher (31 and 36%, respectively). To examine whether the fractionation strategy allowed us to profile intact or partially intact protein complexes, we correlated protein abundances with reported *E. coli* monomeric and native (protein complex) M_r_ values. Supported by clustering data, abundance patterns for more than 120 uncharacterized proteins suggested participation in complexes not characterized to date. In insoluble *E. coli* O157:H7 lysate fractions, high abundances of subunits of large organelle-like assemblies and oligomer-forming proteins previously shown to be prone to aggregation were detected.

## Supporting Information

Figure S1 **Hierarchical clustering with the Pearson correlation metric of STEC proteins with different abundances comparing three size exclusion chromatography fractions - F1-s_SEC_ (+280), F2-s_SEC_ (280-80) and F3-s_SEC_ (80-10 kDa) denoting novel proteins predicted to be part of oligomeric protein complexes.** The software tool MeV was used for the analysis using the Hierarchical Pearson Correlation Metric (average linkage clustering) visualized at the intensity scale of protein abundance APEX_i_ scores from 1.0 to 5000). The soluble cell lysate fractions were F1-sSEC (+280 kDa), F2-sSEC (280-80 kDa) and F3-sSEC (80-10 kDa). Prior to the HCL analysis, 760 proteins of the total EHEC dataset (2521 proteins) observed to be differentially abundant with statistical significance (F1-sSEC vs. F2-sSEC vs. F3-sSEC) at a p-value of <0.02 using the Kruskal Wallis test were selected. In the three clusters, more than 70% of the proteins were part of characterized protein complexes in the approximated M_r_ range.
(PPTX)

Figure S2 **Euclidian distance clustering of EHEC proteins with different abundances in size exclusion chromatography fractions (F1-sSEC to F3-sSEC) compared to the insoluble lysate fraction (F4-p) denoting proteins part of the ribosome, the O-antigen biosynthesis apparatus and cell division/cytoskeletal elements.** The software tool MeV was used for the analysis using the Hierarchical Euclidian Distance Clustering metric (average linkage clustering) visualized at the intensity scale of protein abundance APEX_i_ scores from 1.0 to 5000. The fractions were F1-sSEC (+280 kDa), F2-sSEC (280-80 kDa), F3-sSEC (80-10 kDa) - all from the soluble fraction - and F4-p, the insoluble cell lysate fraction). Prior to the HCL analysis, 802 proteins of the total EHEC dataset (2521 proteins) observed to be differentially abundant with statistical significance comparing F1–F3 with F4-p using the Wilcoxon Rank Sum test (p-values<0.02) were selected. Proteins that are part of the three clusters are also listed in Table 3. The markers (stripes) to the right of the heat maps each denote the position of a protein in the cluster, in the same order as the listed protein names to the right: - blue, left column: ribosome; - brown, middle column: cell division system and cytoskeleton; - green, right column: O-antigen biosynthesis apparatus. The highlighted cluster at the bottom of the image on the right was enriched in particular in proteins part of the ribosome and O-antigen biosynthesis system, the proteins are listed in the order of their appearance in the HCL tree.
(PPTX)

Table S1 **Quantitative reproducibility of shotgun proteomics analyses of STEC strain 86-24.** BR: biological replicate; T1R: technical replicate at size exclusion chromatography stage; T4R: technical replicate at LC-MS/MS stage; STDEV: standard deviation; R^2^: Pearson correlation coefficient; FDR: protein false discovery rate determined with Mascot algorithm.
(XLS)

Table S2 **Shotgun proteomics analysis of STEC strain 86-24 cell lysates using the modified spectral counting method APEX.** Worksheet A - 52 Fractions_Mascot (1% FDR). Theme colors used in worksheet: pO157 plasmid-encoded proteins (orange); proteins encoded by prophage genome regions (green and, if non-LEE effector proteins, turquoise); non-LEE effectors outside of prophage regions (blue). Column A: GenBank accession number in NCBI - *Escherichia coli* O157:H7 EDL933 protein sequence database (chromosome and pO157 plasmid). Column B: Swiss-Prot accession number - *Escherichia coli* O157:H7 EDL933 protein sequence database (chromosome and pO157 plasmid). Column C: Ref Seq. accession number - *Escherichia coli* O157:H7 EDL933 protein sequence database (chromosome and pO157 plasmid). Column D: Locus tag - *Escherichia coli* O157:H7 EDL933 protein sequence database (chromosome and pO157 plasmid). Column E: Gene name - *Escherichia coli* O157:H7 EDL933 protein sequence database (chromosome and pO157 plasmid). Column F: Replicon - *Escherichia coli* O157:H7 EDL933 genome. Column G: Protein description derived from *Escherichia coli* O157:H7 EDL933 protein sequence database. Column H: APEX_i_ protein quantities (mean averages) from 19 individual 2D-LC-MS/MS experiments using the APEX Quantitative Proteomics Tool (http://www.biomedcentral.com/1471-2105/9/529). The method calculates protein quantities computationally, essentially based on spectral counts, normalizes based on the total number of spectral counts in a given dataset followed by multiplication with an adjustment factor (2.5×10^6^, for the estimated number of protein molecules per cell). Column I-AA: APEX_i_ protein quantities from 19 individual 2D-LC-MS/MS experiments using the APEX method. Column AB: Subcellular localization of proteins derived from assignments with the algorithms in PSORTb (http://www.psort.org/psortb/). Column AC: Subcellular localization of proteins derived from assignments with the algorithms in Cell PLoc (Chou & Shen, Nature Protocols 3, p. 153–162; 2008. Column AD: protein sequence length (in amino acids). Column AE-AI: Subcellular localization of proteins derived from individual bioinformatics searches. Each search parameter is incorporated in the Cell-Ploc tool. Column AJ: Subcellular localization of proteins derived from assignments with the algorithms in Sosui from Gram-negative bacteria (http://bp.nuap.nagoya-u.ac.jp/sosui/). Column AK: Prediction of a type III secretion system component or effector using the Database for type III secretion systems (DTTSS, sdbi.sdut.edu.cn/ttss/). Column AL: Main functional role category based in data in the CMR database (JCVI); selection for *E. coli* EDL933: http://cmr.jcvi.org/tigr-scripts/CMR/GenomePage.cgi?org=ntec02. Column AM: Sub functional role category based in data in the CMR database (JCVI); selection for *E. coli* EDL933: http://cmr.jcvi.org/tigr-scripts/CMR/GenomePage.cgi?org=ntec02. Color codes: pO157 plasmid-encoded proteins (orange); proteins encoded by prophage genome regions (green); if these were predicted non-LEE effectors (turquoise); non-LEE effectors not in prophage regions (blue). Worksheet B - 52 Fractions_InsPecT (0.2% FDR). Column A: GenBank accession number in NCBI - *Escherichia coli* O157:H7 EDL933 protein sequence database (chromosome and pO157 plasmid). Column B: Swiss-Prot accession number - *Escherichia coli* O157:H7 EDL933 protein sequence database (chromosome and pO157 plasmid). Column C: Ref Seq. accession number - *Escherichia coli* O157:H7 EDL933 protein sequence database (chromosome and pO157 plasmid). Column D: Locus tag - *Escherichia coli* O157:H7 EDL933 protein sequence database (chromosome and pO157 plasmid). Column E: Gene name - *Escherichia coli* O157:H7 EDL933 protein sequence database (chromosome and pO157 plasmid). Column F: Replicon - *Escherichia coli* O157:H7 EDL933 genome. Column G: Protein description derived from *Escherichia coli* O157:H7 EDL933 protein sequence database. Column H: Protein scoring was performed with the algorithms InsPecT and MS-GF (http://proteomics.ucsd.edu/Software/MSGeneratingFunction.html). APEX_i_ protein quantities (mean averages) from 19 individual 2D-LC-MS/MS experiments were determined uploading the MS-GF rescored datasets (identified at the 0.2% FDR) into the APEX Quantitative Proteomics Tool (http://www.biomedcentral.com/1471-2105/9/529) and using the APEX method. The method calculates protein quantities computationally essentially based on spectral counts, normalizes based on the total number of spectral counts in a given experiment followed by multiplication with an adjustment factor (2.5×10^6^, for the estimated number of protein molecules per cell). Column I-AA: APEX_i_ protein quantities from 19 individual 2D-LC-MS/MS experiments using the APEX method. Column AB: Subcellular localization of proteins derived from assignments with the algorithms in PSORTb (http://www.psort.org/psortb/). Column AC: Subcellular localization of proteins derived from assignments with the algorithms in Cell PLoc (Chou & Shen, Nature Protocols 3, p. 153–162; 2008. Column AD: protein sequence length (in amino acids). Column AE-AI: Subcellular localization of proteins derived from individual bioinformatics searches. Each search parameter is incorporated in the Cell-Ploc tool. Column AJ: Subcellular localization of proteins derived from assignments with the algorithms in Sosui from Gram-negative bacteria (http://bp.nuap.nagoya-u.ac.jp/sosui/). Column AK: Prediction of a type III secretion system component or effector using the database for type III secretion systems (DTTSS, sdbi.sdut.edu.cn/ttss/). Column AL: Main functional role category based in data in the CMR database (JCVI); selection for *E. coli* EDL933: http://cmr.jcvi.org/tigr-scripts/CMR/GenomePage.cgi?org=ntec02. Column AM: Sub functional role category based in data in the CMR database (JCVI); selection for *E. coli* EDL933: http://cmr.jcvi.org/tigr-scripts/CMR/GenomePage.cgi?org=ntec02.
(XLS)

Table S3 **STEC O157:H7 proteins/peptides identified by LC-nESI-MS/MS on a Linear Ion Trap instrument at a 1% protein false discovery rate.** A: gi| accession numbers are equivalent to those listed in Table S2, Supplemental Information (genome E. coli O157:H7, strain EDL933); B: highest Mascot score for a given protein in the dataset at a 1% FDR; C: .dat file from Mascot search result; D: amino acid sequence for peptide-spectral match; E: start and end amino acid positions within protein sequence as annotated for the respective ORF in the E. coli EDL933 protein sequence database; F: peptide ion score in tandem MS/MS experiment, peptide fragment ions with scores <20 are not listed; G: peptide mass value; H: peptide identification output file.
(XLS)

Table S4 **Shotgun proteomics analysis of STEC strain 86-24 using the modified spectral counting method APEX comparing protein profiles in four lysate fractions.** Worksheet A: F1-sSEC samples, proteins identified in the 13 SCX fractions (F1–13) derived from the first size exclusion chromatography fraction of the soluble cell lysate. Worksheet B: F2-sSEC samples, proteins identified in the 13 SCX fractions (F14–26) derived from the second size exclusion chromatography fraction of the soluble cell lysate. Worksheet C: F3-sSEC samples, proteins identified in the 13 SCX fractions (F27–39) derived from the third size exclusion chromatography fraction of the soluble cell lysate. Worksheet D: F4-p samples, proteins identified in the 13 SCX fractions (F40–52) derived from the insoluble cell lysate fraction. General descriptions for all worksheets: Column A: GenBank accession number in NCBI - *Escherichia coli* O157:H7 EDL933 protein sequence database (chromosome and pO157 plasmid). Column B: Swiss-Prot accession number - *Escherichia coli* O157:H7 EDL933 protein sequence database (chromosome and pO157 plasmid). Column C: Ref Seq. accession number - *Escherichia coli* O157:H7 EDL933 protein sequence database (chromosome and pO157 plasmid). Column D: Locus tag - *Escherichia coli* O157:H7 EDL933 protein sequence database (chromosome and pO157 plasmid). Column E: Gene name - *Escherichia coli* O157:H7 EDL933 protein sequence database (chromosome and pO157 plasmid). Column F: Subcellular localization of proteins derived from assignments of a combination of algorithms to predict subcellular localizations - PSORTb (http://www.psort.org/psortb/), Cell PLoc (Chou & Shen, Nature Protocols 3, p. 153–162; 2008), the Ecocyc database (http://ecocyc.org/) and SwissProt; Protein complex assignments in homooligomeric complexes (e.g. ‘Ho-2’) or heterooligomeric complexes (e.g. ‘Ht-7’, if subunit score unknown ‘Ht-Mu’) were made from data in Ecocyc and Swiss-Prot, if available. Abbr.: CY, cytoplasmic; IM, inner membrane; OM, outer membrane; L, lipoprotein; PP, periplasmic; Ex, extracellular; PP or CY/IM, IM/OM periphery; 50S and 30S Rib, ribosome or ribosome subunit; TMD: transmembrane domain; larger cellular assemblies (ribosome, degradosome, cell divisome) may be experimentally unproven. In worksheet D, the symbol for aggregate formation is AGG. Column G: protein description from EDL933 genome database or, if more information, from Ecocyc and Swiss-Prot databases. Column H: APEX_i_ protein abundance ranking: this data is based on the mean averages of spectral counts derived from 12 individual 2D-LC-MS/MS experimental replicates using the APEX quantitative proteomics tool. The method calculates protein quantities via computationally adjusted spectral counts, normalizes based on the total number of spectral counts in a given experiment followed by multiplication with an adjustment factor (2.5×10^6^, for the estimated number of protein molecules per cell); the protein abundances are ranked using the total number of proteins identified at a 5% protein FDR confidence level. Column I: molecular weight in kDa based on protein sequence annotated in the EDL933 genome. Column J-U: APEX_i_ protein quantities from 12 individual 2D-LC-MS/MS experiments using the APEX method (11 experiments in the case of fraction F4-p). Column V: absolute APEX_i_ protein quantities averaged from 12 replicate 2D-LC-MS/MS experiments using the APEX method (11 experiments in the case of fraction F4-p). Column W: Subcellular localization of proteins derived from assignments with the algorithms in PSORTb (http://www.psort.org/psortb/). Column X: Subcellular localization of proteins derived from assignments with the Cell PLoc tool (Chou & Shen, Nature Protocols 3, p. 153–162; 2008). Column Z-AE: Individual assignments using individual algorithms components of Cell PLoc. Column AF: Prediction of a type III secretion system component or effector using the Database for type III secretion systems (DTTSS, sdbi.sdut.edu.cn/ttss/). Column AG: Main functional role category based in data in the CMR database (JCVI); selection for E. coli EDL933: http://cmr.jcvi.org/tigr-scripts/CMR/GenomePage.cgi?org=ntec02. Column AH: Sub functional role category based in data in the CMR database (JCVI); selection for E. coli EDL933: http://cmr.jcvi.org/tigr-scripts/CMR/GenomePage.cgi?org=ntec02. Specific descriptions for worksheets: Worksheet A: color codes. Orange, proteins experimentally shown to be localized in *E. coli* protein complexes and with the appropriate Mr range of >280 kDa in fraction F1-sSEC; blue, peripheral membrane proteins experimentally shown to be localized in membrane-associated protein complexes (inner or outer membrane); yellow, proteins not experimentally shown to be localized in E. coli complexes, but with protein sequence-based Mr values well below the Mr range of 280 kDa assigned to proteins in fraction F1-sSEC. Worksheet B: color codes. Orange, proteins experimentally shown to be localized in E. coli protein complexes and with an appropriate Mr range of 280-80 kDa in fraction F2-sSEC; yellow, proteins not experimentally shown to be localized in E. coli complexes, but with protein sequence-based Mr values well below the Mr range from 280 to 80 kDa assigned to proteins in fraction F2-sSEC. Worksheet C: color codes. Yellow, periplasmic and extracellular proteins in the 80-10 kDa in fraction (F3-sSEC); many periplasmic proteins appear not to be part of oligomeric complexes according to this data; gray, proteins experimentally shown to be localized in E. coli complexes in Mr ranges above 80 kDa and relatively abundant in fraction F3-sSEC; this data indicates that the protein complexes these protein subunits belong to are not stable under the experimental lysis and fractionation conditions used here. Worksheet D: color codes. Color codes are used to assess the enrichment of certain proteins and protein families in the insoluble cell lysate fraction F4-p: these proteins are part of known or predicted large subcellular protein or protein-ribonucleic acid assemblies; orange, *E. coli* ribosome: ribosomal protein subunits and ribosome-associated proteins; green, other large subcellular assemblies not as well characterized as the ribosome, but with some evidence reported: mRNA degradosome, cell division apparatus, cytoskeletal elements, O-antigen biosynthesis system; glycerol utilization system; blue, subunits of membrane-associated protein complexes; red text in gene name column: cytoplasmic proteins reported to form aggregates or are substrates of chaperones DnaK and GroEL.
(XLS)

Table S5 **Shotgun proteomics analysis of STEC strain 86-24 comparing protein profiles in two insoluble, iodixanol density gradient fractions.** Legends for worksheets A and B. Column A: MS sample ID - mass spectrometry database search file name. Column B: Accession No - gi| protein accession number in NCBI - *Escherichia coli* O157:H7 EDL933 protein sequence database (chromosome and pO157 plasmid). Column C: Protein description - according to protein annotations in NCBI - *Escherichia coli* O157:H7 EDL933. Column D: Mascot protein score - with at least one peptide e-value<0.1. Column E: Total peptides Rank = 1−Rank 1 peptides according to Mascot search results (peptide e-values<0.1). Column F: Protein sequence coverage - according to the Mascot search results (peptide e-values<0.1). Column G: pI - Isoelectric point based on protein sequence annotated in the EDL933 database. Column H: M.W. - Molecular weight in kDa based on protein sequence annotated in the EDL933 database. Column I: TMHMM - Bioinformatic prediction of transmembrane domains with algorithm TMHMM (www.cbs.dtu.dk) to predict integral inner membrane proteins. Column J: LipoP - Bioinformatic prediction of lipoprotein anchor motifs and SpII signal sequences with algorithm LipoP (www.cbs.dtu.dk) to predict lipid-anchored proteins. Column K: Sp I - Bioinformatic prediction of signal cleavage site motifs with algorithm SignalP (www.cbs.dtu.dk) to predict exported (periplasmic/outer membrane) proteins. Column L: PSORTb - Bioinformatic prediction of subcellular localization with the set of algorithms in PSORTb (www.psort.org/psortb/). Column M: Notes about membrane association, aggregation, larger assemblies with protein subunits - to establish protein features causing enrichment in one of the two iodixanol density gradient fractions derived from insoluble EHEC cell lysate matter, we assessed protein biophysical and biochemical traits based on data in the APEX abundance analysis (Table S3) and descriptions for proteins in Ecocyc and SwissProt databases. Abbreviations: iodx_low density worksheet: CY, cytoplasm; IM, cytoplasmic membrane; CY/IM, peripheral membrane protein; Abbreviations: iodx_high density worksheet: categories - ‘aggregation-prone cytoplasmic protein’, ‘membrane protein complex’ (mostly peripheral or monotopic membrane proteins without transmembrane regions), ‘larger assembly’ (larger protein assembly e.g. ribosome, tRNA synthase complex, ‘oligomeric in cytoplasm’ (soluble protein complex in cytoplasm), ‘LP’ (lipoprotein), ‘TMD’ (transmembrane domain proteins), and ‘OM’ (β-barrel outer membrane protein).
(XLS)

Table S6 **Lipoprotein assignments of STEC 86-24 proteins based on enrichment in a size exclusion chromatography fraction of high M_r_ and presence of putative lipid anchor motifs.** Columns A–E, G: accession numbers, locus tags and gene names derived from the annotated genome database of enterohemorrhagic E. coli strain EDL933 (NCBI); gene name in parentheses are from Swiss-Prot or EcoCyc annotations. Column F: data on lipoprotein participation in multi-protein complexes; L: lipoprotein; IM/OM, inner and outer membrane; L?: no predictive information on IM or OM attachment and unusual lipid anchor motifs; Ht-Mu: hetero-multimeric, ‘2’, ‘3’ etc. indicating a defined number of protein subunits in a membrane-localized complex. Columns I/J: APEX_i_ abundance of protein; column I: the proteins identified in each of the three size exclusion chromatography fractions (F1-sSEC, F2-sSEC, F3-sSEC) and the insoluble pellet (Fp-4) were ranked based on their abundance rank within a given fraction; column J: absolute APEX_i_ protein abundance normalized by 2.5×10^6^ protein molecules per cell in fraction F1-sSEC. Column K: The PSORTb prediction for subcellular localization (http://www.psort.org/psortb/). Column L: The LipoP algorithm predicts lipoprotein anchor motifs based on a hydrophobic N-terminal sequence followed by a moderately conserved five-amino acid motif with a cysteine in core position 4. This cysteine is covalently coupled to a phospholipid in the mature lipoprotein (http://www.cbs.dtu.dk/services/LipoP/). Column M: Δseq, apparently incorrect prediction of translational start site in ORF, as annotated in EDL933 genome; the amino acid (aa) number indicated the predicted aa residue shift. Color codes: experimentally verified lipoproteins (yellow); strongly conserved Sp II motif with hydrophobic leader sequence in N-terminal Sp II signal sequence (no color); putative Sp II motif for a protein enriched in F1-sSEC fraction (gray). Color themes, worksheet A. Yellow, outer membrane proteins including outer membrane β-barrel and lipoproteins; blue, inner membrane proteins, including transmembrane domain and lipoproteins; gray, peripheral inner membrane proteins and non-membrane contaminant proteins. Color themes, worksheet B. Yellow, proteins part of membrane protein complexes but without transmembrane domains; orange, proteins part of well or partially characterized larger subcellular assemblies; gray, soluble cytoplasmic proteins denoted in the literature or shown in this study to be prone to aggregation or subunits of cytoplasmic protein complexes. The three categories may overlap to some extent.
(XLS)

Table S7 **Statistical evaluation of STEC strain 86-24 protein enrichment patterns in different size exclusion chromatography and cell lysate fractions.** Legend for worksheet A (KW_F123_p02): Correlation of protein molecular weights and their relative abundances in size exclusion chromatography fractions F1-sSEC, F2-sSEC, F3-sSEC. Column A: protein ID in Multiple Experiment Viewer (MeV) experiment. Column B: descriptive protein input in Multiple Experiment Viewer (MeV) experiment. Column C: GenBank accession number in NCBI - Escherichia coli O157:H7 EDL933 protein sequence database (chromosome and pO157 plasmid). Column D: Locus tag - Escherichia coli O157:H7 EDL933 protein sequence database (chromosome and pO157 plasmid). Column E: Gene name - Escherichia coli O157:H7 EDL933 protein sequence database (chromosome and pO157 plasmid). Column F: Subcellular localization of proteins derived from assignments via a combination of algorithms to predict subcellular localizations - PSORTb (http://www.psort.org/psortb/), Cell PLoc (Chou & Shen, Nature Protocols 3, p. 153–162; 2008), the Ecocyc database (http://ecocyc.org/) and Swiss-Prot; Protein complex assignments in homooligomeric complexes (e.g. ‘Ho-2’) or heterooligomeric complexes (e.g. ‘Ht-7’, if subunit score unknown ‘Ht-Mu’) were made from data in Ecocyc and Swiss-Prot, if available. Abbr.: CY, cytoplasmic; IM, inner membrane; OM, outer membrane; L, lipoprotein; PP, periplasmic; Ex, extracellular; PP or CY/IM, IM/OM periphery; 50S and 30S Rib, ribosome or ribosome subunit. Column G: molecular weight in Da calculated from protein sequence length (in amino acids). Column H: molecular weight in Da calculated from a native protein complex (if subunit composition known); if homooligomeric structure was not known (‘Ho-Mu’), we calculated M.W. for a hexamer; if heterooligomeric structure was not known (‘Ht-‘Mu’), we calculated M.W. from the sum the proteins/subunits; for large assemblies (cytoskeletal elements, cell division septa and ribosomes, we used a large M.W. 999,999 Da by default). Column I: Proteins were ordered based on their average abundance ranking according to APEX_i_ values in each of the fractions F1-sSEC, F2-sSEC, and F3-sSEC. Column J: Proteins isoelectric points. Column K: Protein descriptions in the EDL933 database. Column L-O: Kruskal-Wallis statistical test (three groups, group 1: fractions F1-sSEC; group 2: F2-sSEC; group 3: F3-sSEC); proteins with p-values<0.02 were accepted as statistically significant. Column P-Z, AB-AL, AN-AX: APEX_i_ protein quantities from 11 individual 2D-LC-MS/MS replicates using the APEX method, for each of the fractions F1-sSEC, F2-sSEC, and F3-sSEC. Column AA, AM, AY: Protein quantities (mean averages, fractions F1-sSEC, F2-sSEC and F3-sSEC) from 11 individual 2D-LC-MS/MS experiments using the APEX method. The method calculates protein quantities computationally essentially based on spectral counts, normalizes based on the total number of spectral counts in a given experiment followed by multiplication with an adjustment factor (2.5×10^6^, for the estimated number of protein molecules per cell). Color code (yellow): proteins part of higher order protein complexes judged form the Mr of the analyzed SEC fraction and the monomeric Mr. Legend for worksheet B (KW_F123vsF4_p02): Correlation of protein properties and APEX_i_ abundances in fractions F1-sSEC, F2-sSEC, and F3-sSEC versus insoluble lysate fraction F4-p Column A: protein ID in Multiple Experiment Viewer (MeV) experiment. Column B: descriptive protein input in Multiple Experiment Viewer (MeV) experiment. Column C: GenBank accession number in NCBI - Escherichia coli O157:H7 EDL933 protein sequence database (chromosome and pO157 plasmid). Column D: Locus tag - Escherichia coli O157:H7 EDL933 protein sequence database (chromosome and pO157 plasmid). Column E: Gene name - Escherichia coli O157:H7 EDL933 protein sequence database (chromosome and pO157 plasmid). Column F: Subcellular localization of proteins derived from assignments via a combination of algorithms to predict subcellular localizations - PSORTb (http://www.psort.org/psortb/), Cell PLoc (Chou & Shen, Nature Protocols 3, p. 153–162; 2008), the Ecocyc database (http://ecocyc.org/) and Swiss-Prot; Protein complex assignments in homooligomeric complexes (e.g. ‘Ho-2’) or heterooligomeric complexes (e.g. ‘Ht-7’, if subunit score unknown ‘Ht-Mu’) were made from data in Ecocyc and Swiss-Prot, if available. Abbr.: CY, cytoplasmic; IM, inner membrane; OM, outer membrane; L, lipoprotein; PP, periplasmic; Ex, extracellular; PP or CY/IM, IM/OM periphery; 50S and 30S Rib, ribosome or ribosome subunit. Column G: molecular weight in Da calculated from protein sequence length (in amino acids). Column H: molecular weight in Da calculated from a native protein complex (if subunit composition known); if homooligomeric structure was not known (‘Ho-Mu’), we calculated M.W. for a hexamer; if heterooligomeric structure was not known (‘Ht-‘Mu’), we calculated M.W. from the sum of the proteins/subunits; for large assemblies (cytoskeletal elements, cell division septa and ribosomes, we used a large M.W. 999,999 Da by default). Column I: Proteins were ordered based on their average abundance ranking according to APEX_i_ values in the sum of the fractions F1-sSEC, F2-sSEC, and F3-sSEC versus F4-p. Column J: Proteins isoelectric points. Column K: Protein descriptions in the EDL933 database. Column L-P: Wilcoxon rank sum statistical test (two groups, group 1: fractions F1-sSEC, F2-sSEC, and F3-sSEC; group 2: F4-p); the groups essentially represent the soluble fraction and the insoluble fraction of EHEC cell lysates; proteins with p-values<0.02 were accepted as statistically significant. Column R-AX, AZ-BJ: APEX_i_ protein quantities from 33 and 11 2D-LC-MS/MS experimental replicates for all combined fractions (F1-sSEC, F2-sSEC, F3-sSEC) and the F4-p fraction, respectively. Column AY and BK: Protein quantities (mean averages) from the fraction combinations described above, using the APEX method. The method calculates protein quantities computationally essentially based on spectral counts, normalizes based on the total number of spectral counts in a given experiment followed by multiplication with an adjustment factor (2.5×10^6^, for the estimated number of protein molecules per cell).
(XLS)
