# Supplementary material for: Characterizing the Escherichia coli O157:H7 Proteome Including Protein Associations with Higher Order Assemblies
Source: PLoS One. 2011 Nov 7;6(11):e26554. doi: 10.1371/journal.pone.0026554 (PMC3210124; doi:10.1371/journal.pone.0026554)
Supplement: Figure S1 — Hierarchical clustering with the Pearson correlation metric of STEC proteins with different abundances comparing three size exclusion chromatography fractions - F1-sSEC (+280), F2-sSEC (280-80) and F3-sSEC (80-10 kDa) denoting novel proteins predicted to be part of oligomeric protein complexes. The software tool MeV was used for the analysis using the Hierarchical Pearson Correlation Metric (average linkage clustering) visualized at the intensity scale of protein abundance APEXi scores from 1.0 to 5000). The soluble cell lysate fractions were F1-sSEC (+280 kDa), F2-sSEC (280-80 kDa) and F3-sSEC (80-10 kDa). Prior to the HCL analysis, 760 proteins of the total EHEC dataset (2521 proteins) observed to be differentially abundant with statistical significance (F1-sSEC vs. F2-sSEC vs. F3-sSEC) at a p-value of <0.02 using the Kruskal Wallis test were selected. In the three clusters, more than 70% of the proteins were part of characterized protein complexes in the approximated Mr range. (PPTX) [file pone.0026554.s001.pptx]

## Slide 1
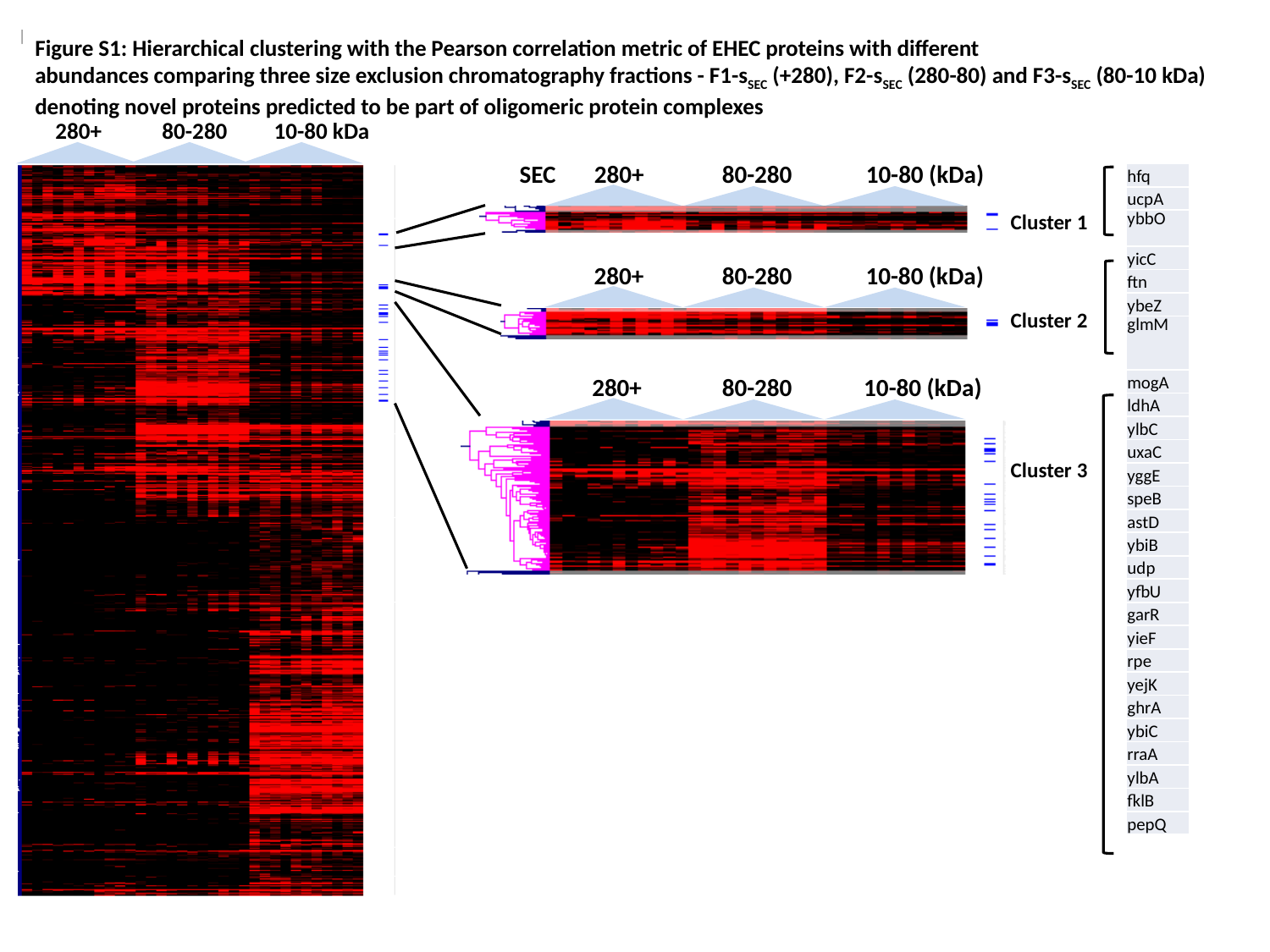

Figure S1: Hierarchical clustering with the Pearson correlation metric of EHEC proteins with different
abundances comparing three size exclusion chromatography fractions - F1-sSEC (+280), F2-sSEC (280-80) and F3-sSEC (80-10 kDa)
denoting novel proteins predicted to be part of oligomeric protein complexes
280+
80-280
10-80 kDa
SEC 280+
80-280
10-80 (kDa)
| hfq |
| --- |
| ucpA |
| ybbO |
| yicC |
| ftn |
| ybeZ |
| glmM |
| mogA |
| ldhA |
| ylbC |
| uxaC |
| yggE |
| speB |
| astD |
| ybiB |
| udp |
| yfbU |
| garR |
| yieF |
| rpe |
| yejK |
| ghrA |
| ybiC |
| rraA |
| ylbA |
| fklB |
| pepQ |
Cluster 1
280+
10-80 (kDa)
80-280
Cluster 2
280+
10-80 (kDa)
80-280
Cluster 3
