# Supplementary material for: Characterizing the Escherichia coli O157:H7 Proteome Including Protein Associations with Higher Order Assemblies
Source: PLoS One. 2011 Nov 7;6(11):e26554. doi: 10.1371/journal.pone.0026554 (PMC3210124; doi:10.1371/journal.pone.0026554)
Supplement: Figure S2 — Euclidian distance clustering of EHEC proteins with different abundances in size exclusion chromatography fractions (F1-sSEC to F3-sSEC) compared to the insoluble lysate fraction (F4-p) denoting proteins part of the ribosome, the O-antigen biosynthesis apparatus and cell division/cytoskeletal elements. The software tool MeV was used for the analysis using the Hierarchical Euclidian Distance Clustering metric (average linkage clustering) visualized at the intensity scale of protein abundance APEXi scores from 1.0 to 5000. The fractions were F1-sSEC (+280 kDa), F2-sSEC (280-80 kDa), F3-sSEC (80-10 kDa) - all from the soluble fraction - and F4-p, the insoluble cell lysate fraction). Prior to the HCL analysis, 802 proteins of the total EHEC dataset (2521 proteins) observed to be differentially abundant with statistical significance comparing F1–F3 with F4-p using the Wilcoxon Rank Sum test (p-values<0.02) were selected. Proteins that are part of the three clusters are also listed in Table 3. The markers (stripes) to the right of the heat maps each denote the position of a protein in the cluster, in the same order as the listed protein names to the right: - blue, left column: ribosome; - brown, middle column: cell division system and cytoskeleton; - green, right column: O-antigen biosynthesis apparatus. The highlighted cluster at the bottom of the image on the right was enriched in particular in proteins part of the ribosome and O-antigen biosynthesis system, the proteins are listed in the order of their appearance in the HCL tree. (PPTX) [file pone.0026554.s002.pptx]

## Slide 1
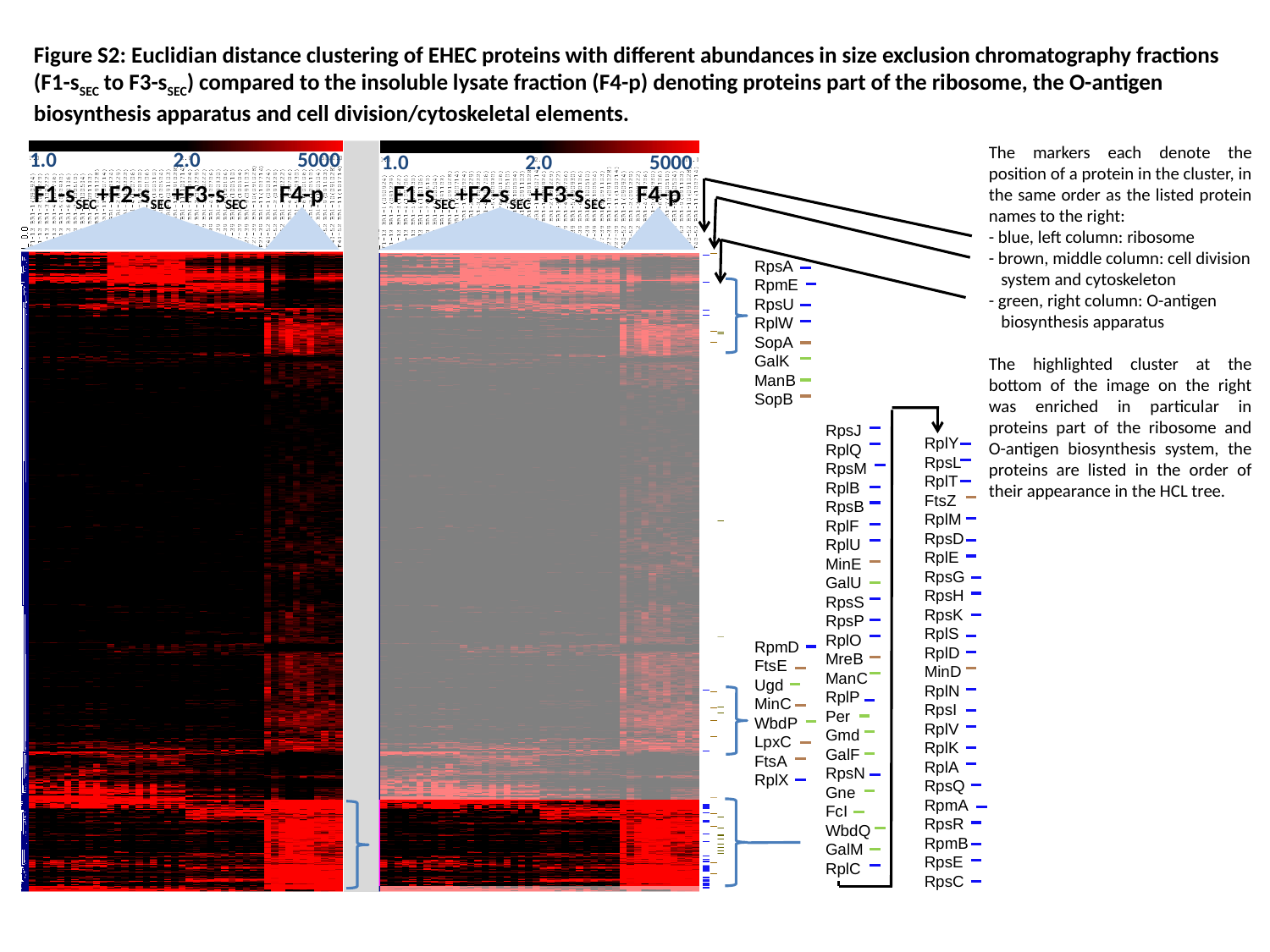

Figure S2: Euclidian distance clustering of EHEC proteins with different abundances in size exclusion chromatography fractions (F1-sSEC to F3-sSEC) compared to the insoluble lysate fraction (F4-p) denoting proteins part of the ribosome, the O-antigen biosynthesis apparatus and cell division/cytoskeletal elements.
The markers each denote the position of a protein in the cluster, in the same order as the listed protein names to the right:
- blue, left column: ribosome
- brown, middle column: cell division
 system and cytoskeleton
- green, right column: O-antigen
 biosynthesis apparatus
The highlighted cluster at the bottom of the image on the right was enriched in particular in proteins part of the ribosome and O-antigen biosynthesis system, the proteins are listed in the order of their appearance in the HCL tree.
1.0 2.0 5000
1.0 2.0 5000
F1-sSEC+F2-sSEC+F3-sSEC
F4-p
F1-sSEC+F2-sSEC+F3-sSEC
F4-p
RpsA
RpmE
RpsU
RplW
SopA
GalK
ManB
SopB
RpmD
FtsE
Ugd
MinC
WbdP
LpxC
FtsA
RplX
RpsJ
RplQ
RpsM
RplB
RpsB
RplF
RplU
MinE
GalU
RpsS
RpsP
RplO
MreB
ManC
RplP
Per
Gmd
GalF
RpsN
Gne
FcI
WbdQ
GalM
RplC
RplY
RpsL
RplT
FtsZ
RplM
RpsD
RplE
RpsG
RpsH
RpsK
RplS
RplD
MinD
RplN
RpsI
RplV
RplK
RplA
RpsQ
RpmA
RpsR
RpmB
RpsE
RpsC
